# EFdA efficiently suppresses HIV replication in the male genital tract and prevents penile HIV acquisition

**DOI:** 10.1128/mbio.02224-22

**Published:** 2023-06-12

**Authors:** Martina Kovarova, Sarah E. Wessel, Claire E. Johnson, Shelby V. Anderson, Mackenzie L. Cottrell, Craig Sykes, Myron S. Cohen, J. Victor Garcia

**Affiliations:** 1 International Center for the Advancement of Translational Science, University of North Carolina at Chapel Hill, Chapel Hill, North Carolina, USA; 2 Division of Infectious Diseases, Department of Medicine, University of North Carolina at Chapel Hill, Chapel Hill, North Carolina, USA; 3 Center for AIDS Research, University of North Carolina at Chapel Hill, Chapel Hill, North Carolina, USA; 4 UNC Eshelman School of Pharmacy, Chapel Hill, North Carolina, USA; 5 Institute for Global Health and Infectious Diseases, University of North Carolina, Chapel Hill, North Carolina, USA; University of California, Davis, California, USA

**Keywords:** human immunodeficiency virus, male genital tract, penile infection, EFdA, HIV prevention, HIV treatment, HIV pathogenesis, stem cells

## Abstract

Sexually transmitted HIV infections in heterosexual men are acquired through the penis. Low adherence to condom usage and the fact that 40% of circumcised men are not protected indicate the need for additional prevention strategies. Here, we describe a new approach to evaluate the prevention of penile HIV transmission. We demonstrated that the entire male genital tract (MGT) of bone marrow/liver/thymus (BLT) humanized mice is repopulated with human T and myeloid cells. The majority of the human T cells in the MGT express CD4 and CCR5. Direct penile exposure to HIV leads to systemic infection including all tissues of the MGT. HIV replication throughout the MGT was reduced 100-1,000-fold by treatment with 4′-ethynyl-2-fluoro-2′-deoxyadenosine (EFdA), resulting in the restoration of CD4^+^ T cell levels. Importantly, systemic preexposure prophylaxis with EFdA effectively protects from penile HIV acquisition.

**IMPORTANCE:**

Over 84.2 million people have been infected by the human immunodeficiency virus type 1 (HIV-1) during the past 40 years, most through sexual transmission. Men comprise approximately half of the HIV-infected population worldwide. Sexually transmitted HIV infections in exclusively heterosexual men are acquired through the penis. However, direct evaluation of HIV infection throughout the human male genital tract (MGT) is not possible. Here, we developed a new *in vivo* model that permits, for the first time, the detail analysis of HIV infection. Using BLT humanized mice, we showed that productive HIV infection occurs throughout the entire MGT and induces a dramatic reduction in human CD4 T cells compromising immune responses in this organ. Antiretroviral treatment with novel drug EFdA suppresses HIV replication in all tissues of the MGT, restores normal levels of CD4 T cells and is highly efficient at preventing penile transmission.

## INTRODUCTION

There are over 38 million people worldwide living with HIV (1). Approximately 1.7 million are newly infected, primarily via sexual transmission (1). Since the beginning of the AIDS epidemic, semen has been recognized as a primary vector of HIV transmission (1). The risk of sexual HIV transmission strongly correlates with the concentration of HIV-RNA in blood and semen (2). HIV transmission is governed by (i) the stage of HIV infection, (ii) CD4^+^ T cell counts, and (iii) the presence of a concurrent sexually transmitted infection (3 - 6). Importantly, in the majority of patients, antiretroviral therapy (ART) rapidly decreases the viral load in blood and semen, and this results in a dramatic reduction in HIV transmission (7). Therefore, treatment as prevention has become an important approach to prevent new HIV infections (7). However, in some individuals with an undetectable plasma viral load, HIV and HIV-infected cells may persist in tissues despite ART (8 - 12).

HIV populations in blood and semen show genetic differences in over 60% of chronically infected men (3, 13, 14). The high degree of HIV compartmentalization between blood and seminal fluids, as well as the oligoclonal amplification that occurs within the seminal tract (13), has been demonstrated using drug resistance markers (15 - 17), population markers (18, 19), and phylogenetic analysis (20 - 24). Viral strains that persist in seminal fluid despite ART have been reported to be distinct from those present in blood (8, 25 - 27). This suggest that HIV in semen may arise from local sources within the male genital tract (MGT), at least in a subset of infected patients. Indeed, viral strains divergent from those found in blood were observed in MGT tissues from chronically infected HIV-positive patients (22, 28) and Simian Immunodeficiency Virus (SIV)-infected macaques (29). The origin of virus present in semen is therefore highly relevant to the implementation of new therapies aimed at effectively reducing HIV transmission.

Previous studies suggested that seminal HIV originates from the prostate or urethra (30, 31). However, recent phylogenetic analyses of SIV populations present in MGT organs and in the peripheral blood of rhesus macaques revealed that the contribution of individual MGT organs to seminal SIV shedding varied among individuals and that it could not be predicted based on the correlation between seminal SIV or proinflammatory cytokine mRNA levels in the different MGT organs (32). Rather than a single source, multiple genital organs are likely involved in the release of virus and infected cells into the semen. This underlines the need for detailed analysis of the individual contributions made by each of the organs comprising the MGT to HIV shedding.

Sexually transmitted HIV infections in exclusively heterosexual men are acquired through the penis, whereas acquisition of HIV in men who have sex with men (MSM) can occur through the rectal or penile epithelium (3). Mucosal epithelia covering the inner foreskin surface and glans have been associated with HIV acquisition due to easily accessible HIV target cells (3). In uncircumcised men, the glans and meatus of the relaxed penis are covered by the foreskin (33). Circumcision has been demonstrated in large randomized clinical trials to reduce the risk of HIV acquisition in men by 50–60% (34, 35). However, studies on HIV infection of the human penis at sites other than the foreskin have been limited by the lack of available penile tissue (36). HIV target cells have been found in the columnar urethral mucosa (37 - 39), the mucosa in the opening of the penile urethra. Limited *in vitro* HIV infection of various penile sites in explants found that the foreskin, glans, meatus, and urethra were all susceptible to infection by CCR5-tropic HIV (39).

Rhesus macaques are often used for evaluation of HIV treatment and prevention strategies (40 - 45). However, the species-specific tropism of HIV prevents the use of transmitted/founder (T/F) viruses and clinical HIV isolates for *in vivo* challenge experiments in non-human primates. In addition, the scarcity of human male genital tract tissues led us to investigate the use of humanized bone marrow/liver/thymus (BLT) mice for (i) the *in vivo* evaluation of penile HIV infection, (ii) the analysis of the effect of antiretroviral therapy on HIV suppression throughout the entire MGT, and (iii) the *in vivo* evaluation of strategies to prevent penile transmission. Humanized BLT mice are immunodeficient mice individually bioengineered to express a *de novo in situ* generated systemic human immune system that allows infection with a variety of transmitted/founder HIV isolates via relevant routes of transmission in humans (46 - 53). Here, we demonstrate that the individual tissues of the MGT in BLT humanized mice are populated by HIV target cells that are susceptible to HIV infection. Direct penile exposure of BLT humanized mice results in systemic HIV infection and CD4^+^ T cell depletion throughout the entire MGT. Treatment with antiretroviral therapy effectively suppresses HIV replication in all compartments of the MGT resulting in the restoration of CD4^+^ T cell levels. Finally, we also demonstrate that preexposure prophylaxis with antiretroviral drugs results in protection from penile HIV infection.

## MATERIALS AND METHODS

### Experimental design

The presence of human hematopoietic cells that are targets of HIV in tissues of the MGT was evaluated by immunohistochemistry. The following tissues were analyzed: prostate, seminal vesicles, epididymis, testes and penile urethra, glans and foreskin, *n* = 6. Cells in tissue sections were stained with specific antibodies against human CD45, CD3, CD4, and CD68 antigens. At least 3 sections of each tissue from each animal were analyzed. Human T cells (CD4^+^ and CD8^+^), macrophages, and cells expressing the CCR5 coreceptor were analyzed using flow cytometry.

The susceptibility of human cells in the MGT to HIV infection was evaluated after intravenous exposure of male BLT mice to HIV-1_JR-CSF_ at 10, 20, and 60 days postexposure (*n* = 4 or 5 per time point). MGT tissues (testes, epididymis, seminal vesicles, prostate, penis) and spleen were collected and analyzed for the presence of cell-associated HIV-RNA and CD4^+^ T cells. The pathogenicity of HIV in the MGT was evaluated as a decrease in the levels of CD4^+^ T cells in tissues compared to the amount of CD4^+^ T cells present early after exposure.

To assess the ability of the transmitted/founder virus HIV-1_CH040_ (54) to infect the MGT of BLT mice, male BLT mice were infected with HIV-1_CH040_ (*n* = 3) intravenously. Mice with established HIV infection were sacrificed, whole epididymis, prostate and seminal vesicles isolated, and cell-associated HIV-RNA and cell-free HIV-RNA levels determined (*n* = 3). In addition, the anatomic location of productively infected cells in the organs of the MGT from infected mice was assessed using RNAscope and immunofluorescence in tissue sections from the testes, epididymis, seminal vesicles, prostate, urethra, glans, and foreskin (at least three sections from each tissue and animal analyzed).

Acquisition of HIV infection via the penis in BLT mice (*n* = 9) after exposure to HIV-1_CH040_ on the meatus urethra was evaluated throughout the course of infection by determining the levels of HIV-RNA in plasma using real-time polymerase chain reaction (PCR) and by measuring the levels of CD4^+^ T cells, CD8^+^ T cells, and T cell activation (CD38^+^HLA-DR^+^) in peripheral blood using flow cytometry. In addition, the levels of the levels of cell-associated viral RNA and DNA in MGT tissues were also analyzed at 5 week postexposure.

The *in vivo* evaluation of the efficacy of antiretroviral drugs was assessed by suppression of HIV infection after daily oral dosing of 4′-ethynyl-2-fluoro-2′-deoxyadenosine (EFdA). Male BLT mice were exposed to HIV-1_JR-CSF_ intravenously. Three weeks postexposure, infected BLT mice were randomly assigned to two groups (*n* = 7), EFdA-treated (1.8 mg/kg, orally daily for 4 weeks) and untreated controls. A Mann–Whitney U-test was performed to ensure that peripheral blood humanization levels (%hCD45 and %hCD4) and HIV-RNA levels in peripheral blood (copies per mL of plasma) between exposure groups were equivalent. A power analysis was performed to determine experimental group sizes needed to achieve 90% power. Following HIV exposure, HIV infection was monitored longitudinally in the peripheral blood of BLT mice by measuring plasma HIV-RNA levels using real-time PCR and CD4^+^ T cell levels by flow cytometry. Animals were harvested at 4 weeks after treatment and cell-associated viral RNA, cell-associated viral DNA, and the relative levels of CD4^+^ T cells in tissues of MGT were evaluated.

For the evaluation of the efficacy of antiretroviral drugs to prevent penile HIV transmission, BLT male mice were treated daily with EFdA (1.8 mg/kg/day). Treated (*n* = 9) and untreated (*n* = 11) animals were exposed to HIV-1_CH040_ via the penis 1, 4, and 8 days after treatment initiation. Two days after the third challenge, EFdA treatment was stopped and HIV-RNA levels in plasma were monitored longitudinally. At the end of the experiment (6 weeks after the first HIV challenge), tissues from the MGT were analyzed for the presence of HIV-DNA. Protection from HIV transmission was statistically determined using a log rank test.

### Preparation of BLT humanized mice

Mice were maintained under specific pathogen-free conditions by the Division of Comparative Medicine at the University of North Carolina–Chapel Hill. BLT humanized mice were prepared as previously described (55). Briefly, a sandwich of human thymus–liver–thymus tissue was implanted under the kidney capsule of irradiated (200 rads) male NSG mice (NOD.Cg-Prkdc^scid^ Il2rg^tm1Wjl^/SzJ, Catalog # 005557, The Jackson Laboratory, Bar Harbor, ME, USA). Following tissue implantation, mice received autologous CD34^+^ hematopoietic stem cells via tail vein injection. Starting at 8 week posttransplantation, human immune cell reconstitution was monitored in the peripheral blood of BLT mice longitudinally by flow cytometry as previously described (55). BLT mice were efficiently reconstituted with a human immune system and used 3–6 months after humanization (6 and 10 months of age) (55). Peripheral blood humanization levels of BLT mice are indicated for each experiment in the result section. Only males were used for experiments due to the focus on the analysis of the MGT.

### Production of HIV stocks and infection of humanized mice

Stocks of HIV-1_JR-CSF_ and HIV-1_CH040_ were generated as previously described and tittered on TZM-bl cells (catalog # 8129, NIH AIDS Research and Reference Reagent Program) to quantify the number of tissue culture infectious units (TCIU)/mL (56 - 58).

To evaluate the ability HIV to infect cells in the MGT, BLT mice were exposed intravenously to 3.0 × 10^4^ TCIU of HIV-1_JR-CSF_ or HIV-1_CHO40_. Penile exposures were performed by placing anesthetized male BLT mice on their back and pipetting the virus inoculum (2 µL) on the penile meatus urethra without protruding the bone out of the penis until the inoculum dissipated into urethra (2 min). This process was repeated until the desired amount of inoculum was administered (< 10 µL or 2 × 10^6^ TCIU). Mice were kept warm on a heating pad until they recovered from anesthesia.

### Immunohistochemical analysis of the MGT of BLT mice

MGT tissues (foreskin, urethra, glans, epididymis, seminal vesicles, prostrate, and testes) were fixed in 10% formalin, paraffin embedded, and cut into 5 µm sections that were mounted onto Superfrost Plus slides (Fisher Scientific, catalog # 12-550-15), incubated at 60°C for 4 hours, deparaffinized with 100% xylene (2 × 3 minutes), and graded ethanol (100% 2 × 3 minutes, 95% 1 × 3 minutes, 90% 1 × 3 minutes, 80% 1 × 3 minutes, 70% ethanol 1 × 3 minutes), then washed in double distilled water. Following antigen retrieval using Diva Decloaker (BioCare Medical, Catalog # DV2004MX) in a 95°C water bath for 30 min, non-specific binding was blocked using Background Sniper (BioCare Medical) for 20 min at room temperature. Sections were incubated at 4°C overnight with the following primary antibodies: monoclonal mouse anti-human CD45 clones 2B11 + PD7/26 (Dako, Catalog # M0701, Lot # 20002173), monoclonal mouse anti-human CD68 Clone KP1 (Dako, Catalog # Mo814, Lot # 20072689), monoclonal rabbit anti-human CD3 clone SP7 (Thermo Fischer Scientific, Catalog # MA1-90582, Lot # 2776351), monoclonal rabbit anti-human CD4 clone SP35 (GenWay, Catalog # 20-786-255112, Lot # 151109LVAC). Tissue sections were also incubated with the following isotype negative control antibodies: negative control mouse IgG1 (Dako, Catalog # X0942, Lot # 00058064) and negative control rabbit IgG (Dako, Catalog # X0936, Lot # 0051308)]. Slides were then washed twice in 1 × TBST wash buffer (5% 1M Tris-HCL PH: 7.4, 0.6% NaCl, 0.05% Tween20 in double distilled water) and endogenous peroxidases were blocked with hydrogen peroxide for 10 min at room temperature. The slides were then washed with 1 × TBST wash buffer (2 × 5 minutes) and developed with the species appropriate MACH polymer system (Mouse MACH 3 Probe, BioCare Medical, Catalog # MP53OH, Lot # 091119-2, Mouse MACH 3 HRP Polymer, BioCare Medical, Catalog # MP53OH, Lot # 091119-2, Rabbit MACH 3 Probe BioCare Medical, Catalog # RH531H, Lot # 041020A, Rabbit MACH 3 HRP Polymer BioCare Medical, Catalog # RH531H, Lot # 041020A) and 3,3′-diaminobenzidine (DAB) chromogen substrate (Vector Laboratories, catalog # SK-4105, Lot ZH0306). Sections were then counterstained with Mayer’s Hematoxylin for 10 min at room temperature. Tissue sections were then washed in double distilled water for 30 min, dehydrated in graded ethanol (70% 1 × 5 minutes, 80% 1 × 5 minutes, 90% 1 × 5 minutes, 95%1 × 5 minutes, and 100% ethanol 2 × 5 minutes) and 100% xylene (2 × 5 minutes), and mounted using Cytoseal XYL (Thermo Scientific, Catalog # 8312-4, Lot # 102708T). Tissue sections were imaged using a Nikon Eclipse Ci microscope using Nikon Elements BR software (version 4.30.01) with a Nikon Digital Sight DS-Fi2 camera.

### Analysis of human immune cell reconstitution in the MGT of BLT mice by flow cytometry

The levels of human immune cells in BLT mice were monitored longitudinally in peripheral blood and at necropsy in the MGT (penis, seminal vesicles, epididymis, prostate, and testes). Briefly, the blood or cells were blocked with 1 mg/mL mouse IgG block (Equitech-Bio, Catalog # SLM56) for 10 min. Cells were incubated with primary antibodies for 30 min in the dark. The following antibodies were used: APC Mouse Anti-Human CD45 (BD Bioscience, Catalog # 555485, Lot # 8338550, Clone HI30), FITC Mouse Anti-Human CD3 (BD Bioscience, Catalog # 555339, Lot # 8151581, Clone HIT3a), APC-H7 Mouse Anti-Human CD4 (BD Bioscience, Catalog # 560158, Lot # 0213417, Clone RPA-T4), PE Mouse Anti-Human CD11b/Mac-1 (BD Bioscience, Catalog # 555388, Lot # 7017973, Clone ICRF44), PerCP Mouse Anti-Human CD8 (BD Bioscience, Catalog # 347314, Lot # 0023245, Clone SK1), PE-Cy 7 Mouse Anti-Human CD45 (BD Bioscience, Catalog # 557835, Lot # 0199128, Clone SJ25C1), FITC Mouse Anti-Human CD8 (BD Bioscience, Catalog # 340692, Lot # 0169506, Clone SK1), PE Mouse Anti-Human CD184 (BD Bioscience, Catalog # 555974, Lot # 8086757, Clone 12G5), PerCP Mouse Anti-Human CD4 (BD Bioscience, Catalog # 347324, Lot # 0045328, Clone SK3), PE-Cy 7 Mouse Anti-Human CD3 (BD Bioscience, Catalog # 557851, Lot # 9220254, Clone SK7), APC Mouse Anti-Human CD195 (BD Bioscience, Catalog # 550856, Lot # 8192953, Clone 3A9), APC-Cy 7 Mouse Anti-Human CD45 (BD Bioscience, Catalog # 557833, Lot # 7279909, Clone 2D1), PE mouse IgG2a,K Isotype Control (Catalog # 555574, Lot # 5064504, Clone G155-178), and APC mouse IgG2a,K Isotype Control (Catalog # 555576, Lot # 7341678, Clone G155-178). Red blood cells were lysed in blood samples using BD FACS Lysis solution (BD Biosciences, Catalog # 349209, Lot # 0126051) for 5 min followed by two washes with FACS Buffer (PBS, 2% FBS). Samples with cells isolated from MGT were washed once with FACS Buffer. Cells were then resuspended in a paraformaldehyde solution (PBS, 2% PFA [Electron Microscopy Sciences, Catalog # 15712, Lot # 190808]). The data were acquired using a BD FACS Canto flow cytometer (BD Biosciences) with BD FACS Diva software version number 6.1.3 (BD Biosciences) and analyzed with FlowJo software version number 10.6.0 (BD Biosciences).

### Analysis of HIV infection in BLT mice

HIV infection was monitored longitudinally in the peripheral blood of BLT mice by measuring HIV-RNA levels in plasma with a real-time PCR viral load assay (assay sensitivity of 687.1 HIV-RNA copies/mL) and by determining CD4^+^ T cell levels by flow cytometric analysis as previously described (56 - 59). The presence of HIV-DNA and HIV-RNA in tissues (spleen, lymph nodes, bone marrow, human thymus, liver, lung and MGT: penis, prostate, seminal vesicles, epididymis, testes) collected from BLT mice at necropsy was determined by real-time PCR analysis of RNA and DNA extracted from 5 × 10^4^–4 × 10^6^ mononuclear cells (assay limit of detection: 10 copies for cell associated DNA and five copies for cell associated RNA). Mononuclear cells were isolated as previously described (55). Briefly, mononuclear cells from the human thymus, lymph nodes, spleen, prostate, seminal vesicles, epididymis, and testes were isolated by passing tissue through a cell strainer. Bones were crushed with a mortar and pestle and cells and tissue fragments passed through a cell strainer to isolate bone marrow cells. The lung, liver and penis were cut into small pieces, digested in an enzyme cocktail containing collagenase D and DNase for 30 min at 37°C and then passed through a cell strainer. Mononuclear cells were isolated from lung and liver using a 40–70% Percoll gradient. Residual red blood cells were lysed with an ammonium/chloride/potassium buffer (54.4 mM ammonium chloride, 10 mM potassium bicarbonate, and 97.3 µM EDTA tetrasodium salt, Sigma-Aldrich). As a control for the presence of intact DNA extracted from human cells, all samples were tested for the presence of human gamma globin DNA by real-time PCR. To evaluate cell-free HIV-RNA levels in the epididymis, seminal vesicles and prostate, tissues were cut to small pieces and incubated in 1 mL of HBSS buffer with mild agitation for 5 min at room temperature (RT). Tissue debris were separated using a cell strainer and samples cleared by centrifugation (300 × g, 5 min, RT). Supernatants were analyzed for HIV-RNA by real-time PCR, results were calculated per whole organ.

### RNA *in situ* hybridization (RNA-ISH) analysis of HIV infection

RNA-ISH was performed on paraffin-embedded 5 µm sections of BLT mouse MGT tissues (urethra, glans, foreskin, prostate, seminal vesicles, epididymis, and testes) using RNAscope 2.5 HD Detection Reagent-RED Kit according to the manufacturer’s instructions. All chemicals used were purchased from Advanced Cell Diagnostics unless specified otherwise. Tissue sections were mounted on Superfrost Plus microscope slides (Fisher Scientific) and heated at 60°C for 4 hours, deparaffinized in xylene (2 × 5 minutes) and then in 100% ethanol (2 × 2 minutes). Slides were then incubated for 10 min at room temperature with hydrogen peroxide to block endogenous peroxidases, followed by heat-induced epitope retrieval in a 95°C water bath in retrieval reagent for 15 min and dehydrated in 100% ethanol. A hydrophobic barrier was used to encircle the section and the slides were incubated with Protease Plus for 15 min at 40°C for all tissues except the prostate and seminal vesicles which were incubated for 30 min at 40°C in a HybEZ hybridization oven. Sections were rinsed in double distilled water and then incubated with prewarmed HIV-RNA probe (Advanced Cell Diagnostics, Catalog # 416111, Lot # 19070A), Negative Control probe (Advanced Cell Diagnostics, Catalog # 310043, Lot # 18162A), or Positive Control probe (Advanced Cell Diagnostics, Catalog # 470-901, Lot # 19071A) for 2 hours at 40°C. Slides were then washed in wash buffer (2 × 2 minutes), excess liquid removed, and then the RNAscope 2.5 HD Detection Reagent-RED Kit was used to amplify and develop the signal using Fast Red substrate. Slides were counterstained with Gills Hematoxylin (Sigma-Aldrich) and mounted with Ecomount (BioCare Medical). Slides were imaged with a Nikon Eclipse Ci microscope equipped with a Nikon DS-Fi2 High-Definition Color Camera and DS-U3 Controller at 20× and 40× magnification.

### Immunofluorescence analysis of HIV infection

MGT tissues collected from mice were fixed in 10% formalin and paraffin embedded. Following deparaffinization and antigen retrieval (Diva Decloaker), tissue sections were incubated in a 10% normal donkey serum solution containing 0.1% Triton X-100 in 1× phosphate-buffered saline (PBS) to block non-specific binding. Tissue sections were then incubated overnight with primary antibodies at 4°C followed by incubation with fluorescent conjugated secondary antibodies for 1 hour at room temperature (AlexaFluor Plus 594 donkey anti rabbit–Invitrogen A32754 (1:1000), Lot # VB292347, AlexaFluor 647 donkey antimouse–Invitrogen A31571 (1:1000), Lot # 2045337). Primary antibodies were directed against HIV-1 (HIV p24–Invitrogen MA1-71515 (1:50), Lot # 20068607) and human CD3 (Dako A0452 (1:50), Lot # GR3274111-3). Background autofluorescence was then quenched with a 0.1% Sudan Black B solution in 80% ethanol prior to staining with 4′,6-diamidino-2-phenylindole (DAPI) (DAPI– ThermoScientific 62248 (1:2000), Lot # VL3159742 and VA2929781). Slides were mounted (ProLong Glass–Invitrogen P36984, Lot # 2273734 and 2210321) and then imaged using an Olympus BX61 upright wide-field microscope using Volocity software (version 6.3) with a Hamamatsu ORCA RC camera. Appropriate negative controls without primary antibodies were also imaged using the same exposure time as matched stained sections. Whole image contrast, brightness, and pseudocoloring were adjusted using ImageJ/Fiji (version 2.0.0-rc-69/1.51 w) and Adobe Photoshop (version CS6).

### EFdA administration and analysis

EFdA (kindly provided by Dr. Parniak, University of Pittsburgh School of Medicine) was reconstituted in sterile PBS at a concentration of 1 mg/mL. EFdA (1.8 mg/kg) was administered to mice once daily by oral gavage. Peripheral blood was collected from animals with capillary tubes coated with or without EDTA to isolate plasma or serum, respectively.

For PK analysis, NSG mice (*n* = 6) were administered EFdA orally once a day and samples collected at 4 hour and 24 hour after the first and seventh doses. The EFdA concentration in samples collected at 4 hour and 24 hour post dosing at day 7 were considered the steady state concentration. In the suppression experiment, EFdA was administered daily and samples were collected at 24 hour postdose at the indicated days. All samples were stored at −80°C until analysis.

Quantification of EFdA plasma concentrations were performed by protein precipitation and LC–MS/MS analysis by the UNC Clinical Pharmacology and Analytical Chemistry Core. Calibration standards and quality control (QC) samples were prepared by diluting the EFdA stock (described above) in blank mouse plasma. Seven microliters of each stored plasma sample were mixed with 35 µL of methanol containing isotopically labeled internal standard (zidovudine-d_4_). Following vortex and centrifugation steps, 25 µL of the resulting supernatant was mixed with 30 µL of water prior to LC–MS/MS analysis. EFdA was eluted from a Waters Atlantis T3 (2.1 × 50 mm, 3 µm particle size) analytical column. An API 5000 triple quadrupole mass spectrometer (AB Sciex, Foster City, CA, USA) was used to detect the analyte. The data were collected using AB Sciex Analyst Chromatography Software with a dynamic range of 0.2–500 ng/ml. Calibration curves were obtained using a weighted (1 /x*x) linear regression of analyte:internal standard peak area ratio vs nominal concentration. All calibration standards and QCs were within 20% (25% at the lower limit of quantification) of the nominal value.

### Statistical analysis

A two-sided Mann–Whitney U-test was used to compare human immune cell levels and CCR5 expression in the MGT tissues of uninfected male BLT mice (Fig. 3). A Kruskal–Wallis test with Dunn’s multiple comparisons test were used to compare the levels of cell-associated HIV-RNA and CD4^+^ T cells in individual MGT tissues of BLT mice at different time points post HIV infection (Fig. 5). A two-sided Mann–Whitney U-test was also used to compare the levels of cell-associated HIV-RNA, cell-associated HIV-DNA, and CD4^+^ T cell levels in the MGT of untreated and EFdA-treated HIV-infected BLT mice as well as the EFdA plasma concentration after the first dose and during steady state (Fig. 7). A log rank (Mantel–Cox) test was used to compare protection from HIV infection between EFdA-treated and untreated animals (Fig. 8). All statistical analyses were performed using GraphPad Prism software (version 9) (La Jolla, CA, USA).

## RESULTS

### Human immune cells efficiently populate the MGT

**Fig 1:**
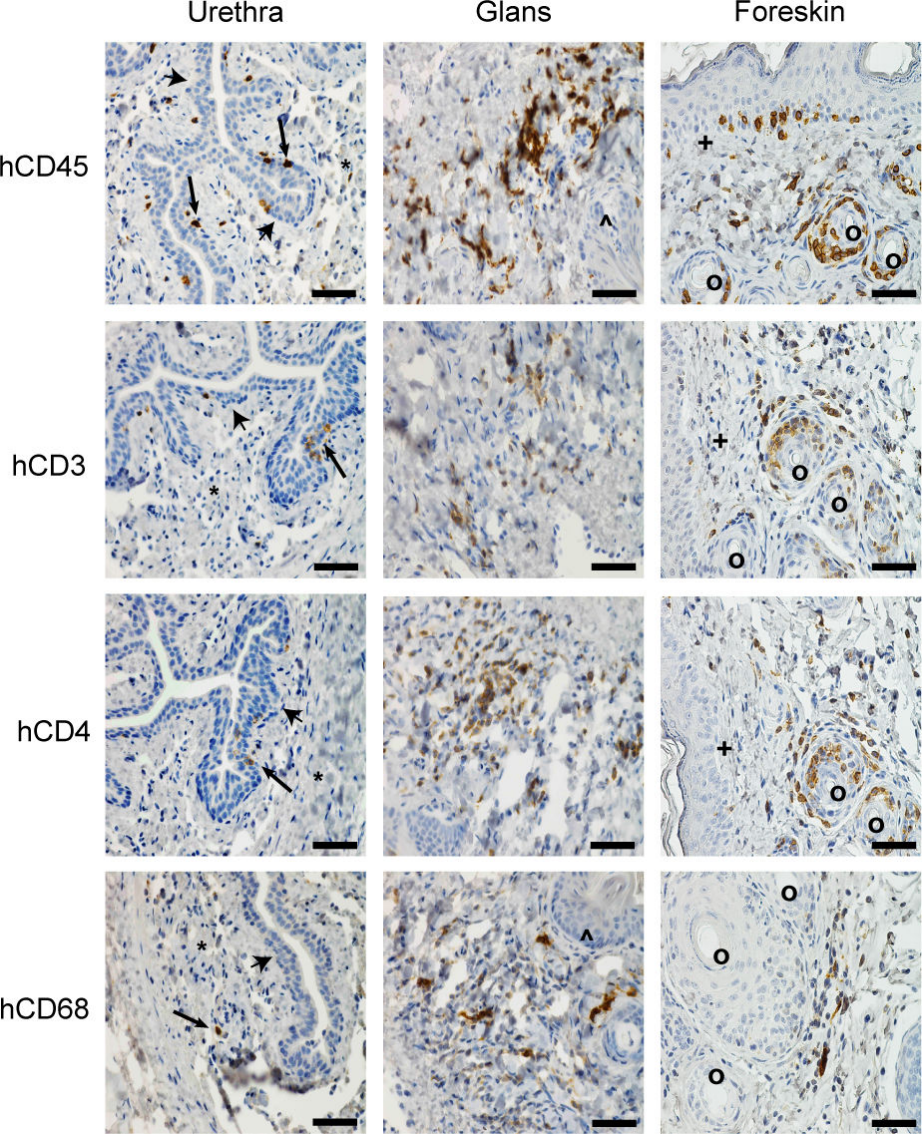
Human hematopoietic cells relevant to HIV infection efficiently populate the penis. Immunohistochemical analysis of the hematopoietic cell subsets (CD45^+^ human cells, CD3^+^ T cells, CD4^+^ T cells, CD68^+^ myeloid cells) present in the penile tissues (urethra, glans, and foreskin) of a male BLT mouse. Positive cells are stained dark brown (arrows) and nuclei are stained blue. Urethral epithelium (arrowheads), lamina propria (*), keratinized penile spines (^), foreskin epithelium (+), hair follicles in foreskin (°). Scale bars represent 50 µm. Tissue sections shown are representative of 12 sections taken from six uninfected BLT mice (*n* = 6).

**Fig 2:**
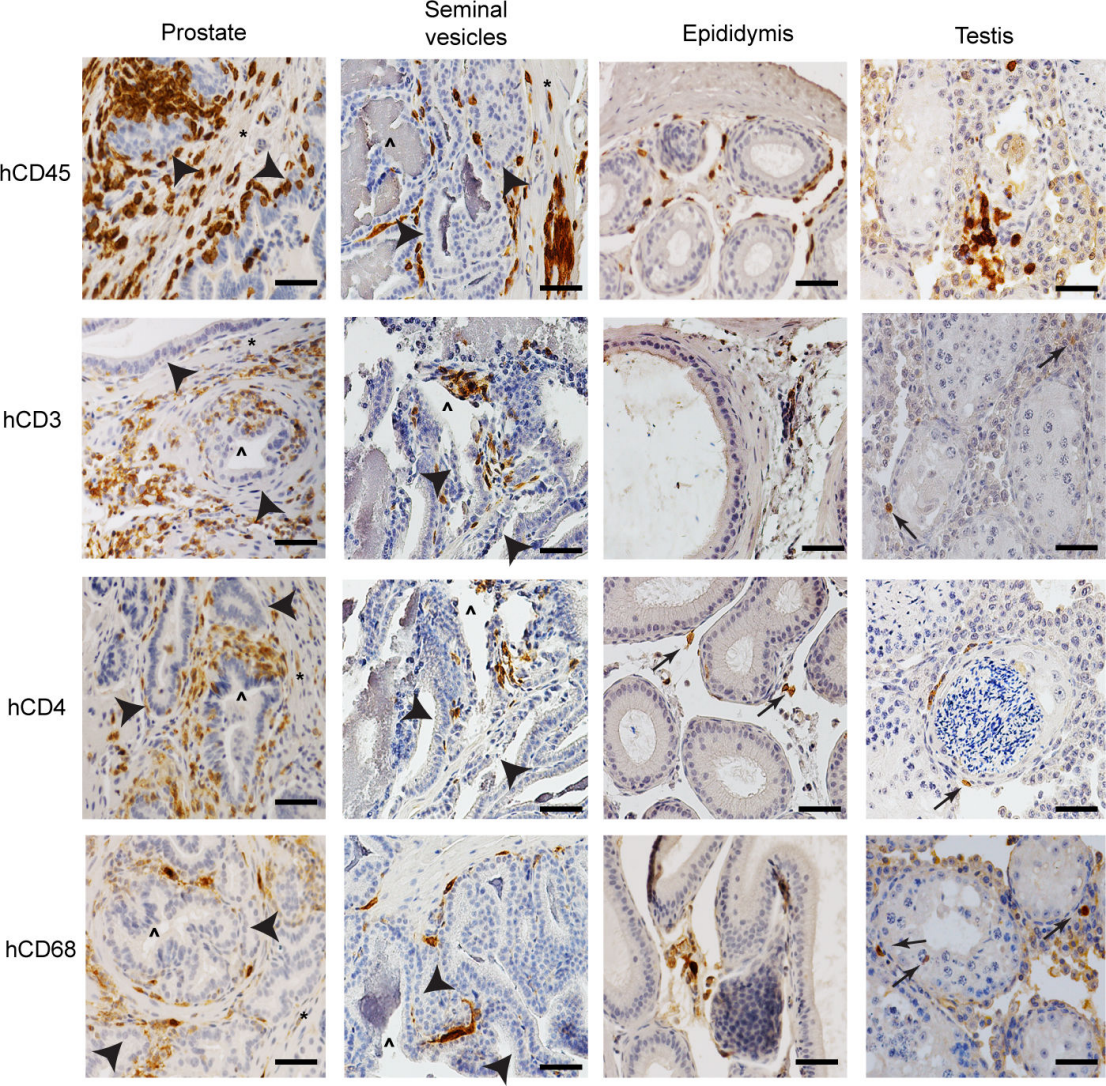
Human hematopoietic cells are present throughout the entire male genital tract. Hematopoietic cell subsets (CD45^+^ human cells, CD3^+^ T cells, CD4^+^ T cells, and CD68^+^ myeloid cells) present in the male genital tract tissues (prostate, seminal vesicles, epididymis, and testis) of BLT mice. Positive cells are stained dark brown; nuclei are stained blue. Epithelium (arrowheads), smooth muscle (*), gland lumen (^). Scale bars represent 50 µm. Tissue sections shown are representative of 12 sections taken from six uninfected BLT mice. *n* = 6.

Humanized BLT mice were constructed as previously described (46 - 53). Reconstitution with human hematopoietic cells (CD45^+^) in peripheral blood was determined by flow cytometric analysis (median 28.5%, range 14.1–64.0%, interquartile range 20.3–50.3%, *n* = 6). The presence and distribution of human CD4^+^ T cells and macrophages, the main targets for HIV infection, were evaluated in the tissues comprising the MGT using immunohistochemistry (IHC). Human CD45^+^, CD3^+^, CD4^+^, and CD68^+^ cells were found in the penis, prostate, seminal vesicles, epididymis, and testes (Fig. 1 and 2; Fig. S1 and S2). Human CD3^+^ and CD4^+^ cells in the penile urethra were predominantly located in close proximity to the urethral epithelium as single cells or as focal aggregates, whereas human CD68^+^ cells were spread throughout the lamina propria (Fig. 1). Human CD3^+^, CD4^+^, and CD68^+^ cells in the penile glans were found in small cell clusters or as scattered single cells in close proximity to the glans surface near keratinized penile spines (Fig. 1). In the foreskin, human cells (CD3^+^, CD4^+^, and CD68^+^ cells) were found in close proximity to the epithelium. CD3^+^ and CD4^+^ cells were also found around hair follicles (Fig. 1). In the prostate of BLT mice, human cells were observed in the columnar epithelium surrounding the prostate glands and in clusters in the tissue stroma (Fig. 2). In the seminal vesicles, human cells were found in the pseudostratified columnar epithelium of highly branched folds of the lumen and in the smooth muscle tissue that surrounds the gland (Fig. 2). In the epididymis, human CD3^+^, CD4^+^, and CD68^+^ cells were found in the epithelial layer and in the smooth muscle (Fig. 2). Human T cells and macrophages were found in clusters located within the interstitium (Fig. 2). In summary, our analysis of the MGT of BLT mice indicates that HIV target cells are distributed throughout all the individual components of the MGT, and their distribution resembles that of immune cells in healthy human MGT (3). Together, these data establish the efficient repopulation and appropriate distribution throughout the entire MGT of the human immune cells known to be targets of HIV infection in humans.

### Human T cells in the MGT express CCR5, the major HIV coreceptor required for HIV mucosal transmission

**Fig 3:**
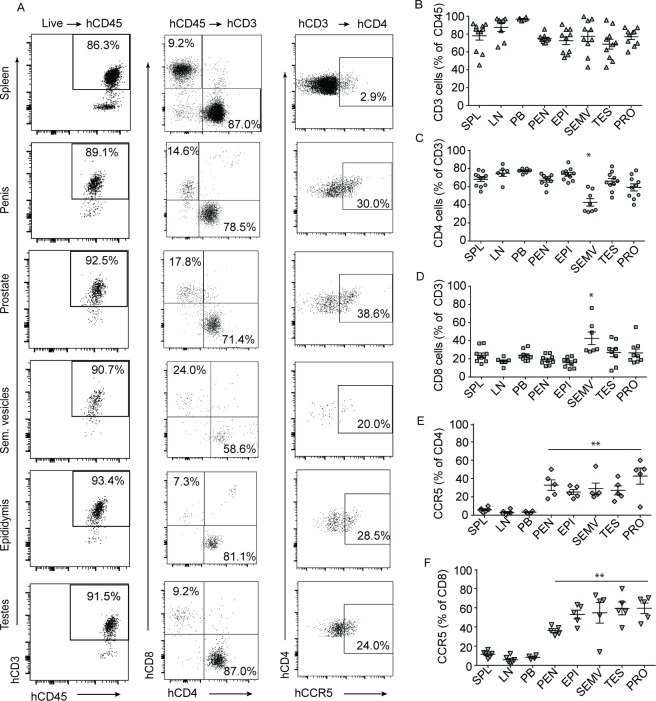
Flow cytometry analysis of hematopoietic cells present in the male genital tract. (**A**) Cells isolated from the penis, prostate, seminal vesicles, epididymis, testes, and spleen of a representative male BLT mouse analyzed by flow cytometry to demonstrate reconstitution with human CD4^+^ T cells, CD8^+^ T cells, and CCR5^+^ CD4^+^ T cells in each compartment. T cells are shown as a percentage of the CD45 human hematopoietic cells present (**B**) and CD4^+^ T cells (**C**) and CD8^+^ T cells (**D**) are shown as a percentage of the CD3^+^ T cells in the penis, prostate, seminal vesicles, epididymis, testes, spleen, lymph nodes (LN), and peripheral blood (PB). Analysis of CCR5 expression on CD4^+^ T cells (**E**) and CD8^+^ T cells (**F**) in MGT tissues, spleen, LN, and PB. Lines show the mean for each tissue. A Mann–Whitney U-test was used to compare the frequencies of immune cell populations between the spleen and MGT tissues of BLT mice. SPL spleen, LN lymph nodes, PB peripheral blood, PEN penis, EPI epididymis, SEMV seminal vesicles, TES testis, and PRO prostate. *P < 0.05, **P < 0.01, *n* = 6.

Flow cytometric analysis was used to show that the majority of the hematopoietic cells present throughout the MGT are human T cells (CD45^+^CD3^+^) (Fig. 3A and B). Of the human T cells present in the MGT, the majority (60–80%) were CD4^+^ (CD45^+^CD3^+^CD4^+^) (Fig. 3C and D). In addition, macrophages were detected in all of the MGT tissues albeit at low levels (CD45^+^CD3^-^CD19^-^CD11b^+^, Fig. S3). The majority of the human T cells present in the MGT of BLT mice express CCR5 (Fig. 3E and F; Fig. S4) (60, 61). CCR5 expression levels were significantly higher on T cells isolated from the MGT compared to T cells isolated from the peripheral blood, lymph nodes, and spleen (CCR5 expression on human CD4^+^ T cells compared to the spleen: penis *P* = 0.0043, epididymis *P* = 0.0043, seminal vesicles *P* = 0.0043, testes *P* = 0.0043, prostate *P* = 0.0087; for CD8^+^ T cells: penis *P* = 0.0043, epididymis *P* = 0.0043, seminal vesicles *P* = 0.0087, testes *P* = 0.0043, prostate *P* = 0.0043). These results are consistent with previous analyses of other human mucosal tissues including the female reproductive tract (FRT) and with the preferential transmission of CCR5-tropic HIV isolates (62, 63).

**Fig 4:**
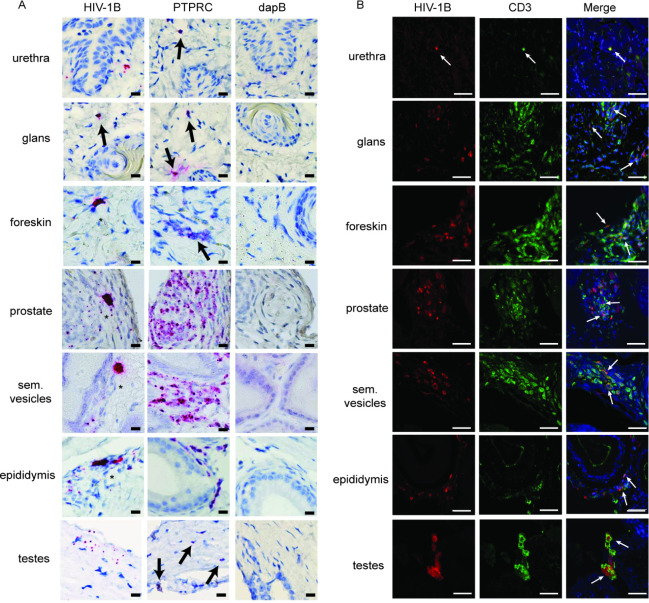
HIV-RNA and p24 expression in the MGT. Male BLT mice were infected intravenously with HIV-1_CH040_ (*n* = 3) (3 × 10^4^ TCIU). Two weeks postinfection mice were euthanized, individual MGT tissues isolated, fixed in 10% formalin, paraffin-embedded, and analyzed by RNAscope and immunofluorescence. (**A**) Analysis of gag HIV-RNA in MGT tissues using RNAscope with the Fast Red colorimetric system. A probe specific for HIV-1 clade B (HIV-1B) was used to identify HIV, a probe specific for human tyrosine phosphatase receptor type C (PTPRC or CD45) mRNA was used to identify human hematopoietic cells. A probe specific for bacterial 4-hydroxy-tetrahydrodipicolinate reductase (or dapB) was used as a negative control. Mayer’s hematoxylin was used as counterstain. Arrows: HIV-infected cells, * clusters of productively infected cells. Scale bars = 25 μm. (**B**) Double immunofluorescence staining analysis for HIV p24 (red) and human CD3 (green) expression. DAPI (shown in blue) was used as counterstain for nucleated cells. Arrows indicate examples of infected T cells. The scale bars represent 50 µm. Sections shown are representative of 6 sections taken from three infected BLT mice.

### Human T cells present in the MGT are susceptible to HIV infection

The susceptibility of the human cells present in the MGT to HIV infection was evaluated in BLT mice (*n* = 6, hCD45^+^ cells: median 44.8%, range 36.3–66.4%). BLT mice were infected intravenously with HIV-1_JRC-SF_ (*n* = 3), an early passage CCR5-tropic primary isolate that has been extensively characterized in this model (64) or with HIV-1_CH040_ (*n* = 3), a CCR5-tropic transmitted/founder virus (3 × 10^4^ tissue culture infectious units (TCIU)) that initiated a natural HIV infection (54). HIV infection was verified in peripheral blood plasma 2 weeks postinfection (HIV-1_JR-CSF_ median 7.6 × 10^6^ HIV-RNA copies/mL, range 2.47–8.63 × 10^6^, HIV-1_CH040_ median 16.62 × 10^6^ HIV-RNA copies/mL, range 3.72–19.50 × 10^6^). Two weeks postinfection, mice were sacrificed and MGT tissues harvested for analysis. *In situ* hybridization with RNAscope was used to evaluate the anatomic location of productively infected cells in the MGT of mice infected with HIV-1_CH040_. HIV-RNA^+^ cells were detected in all MGT tissues from BLT mice (i.e., urethra, glans, foreskin, prostate, seminal vesicles, epididymis, and testes). As shown in Fig. 4A, infected cells typically had a dense HIV gag probe staining signal that encompassed the entire cell body. In multiple tissues, including the foreskin, prostate, seminal vesicles, and epididymis, we found clusters of cells infected with HIV (Fig. 4A, asterisk). Immunofluorescence (IF) staining was then used to identify cells expressing HIV p24 (Fig. 4B). HIV p24^+^ cells were found in all tissues of the MGT. IF costaining for HIV p24 and either human CD3 or human CD68 was then used to demonstrate that the vast majority of the productively infected human cells present in the testes, epididymis, seminal vesicles, prostate, urethra, glans, and foreskin were CD3 positive (Fig. 4B, Fig. S5).

**Fig 5:**
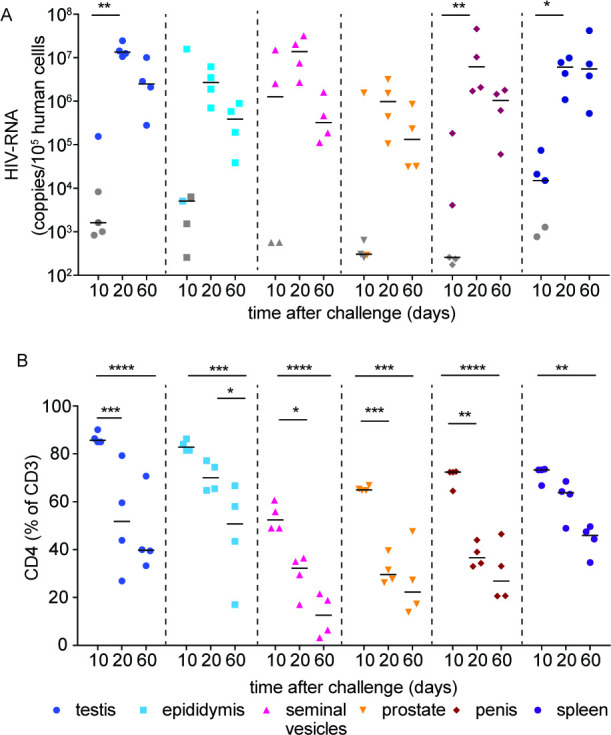
Kinetics of HIV infection and its sequela in the male genital tract. Male BLT mice were infected with HIV-1_JR-CSF_ intravenously (3 × 10^4^ TCIU). At 10, 20, and 60 days postinfection, the testis, epididymis, seminal vesicles, prostate, penis, and spleen were analyzed for cell-associated HIV-RNA (**A**) and CD4^+^ T cells levels (**B**). HIV-RNA limit of detection (LOD) was calculated for each individual sample based on the number of human cells available for analysis. Grey symbols indicate samples below the LOD, lines indicate the median for each data set. A Mann–Whitney U-test was used to compare the frequencies of CD4^+^ T cell levels between samples collected at 10 day postinfection and those present at 20 or 60 day postinfection. *P < 0.05, **P < 0.01, ***P < 0.001, ****P < 0.0001, *n* = 5 at 10 day postinfection, *n* = 4 at 20 and 60 days postinfection.

Semen contains both cell-free and cell-associated virus (3). While cells in semen can originate from any organ of the MGT (3), seminal plasma originates primarily from three organs: seminal vesicles, prostate, and epididymis (65). Therefore, the epididymis, prostate, and seminal vesicles from BLT mice infected with either HIV-1_JR-SCF_ or HIV-1_CH040_ were analyzed individually for the presence of cell-free and cell-associated virus (Fig. S6). Cell-free and cell-associated HIV-RNA was found in the epididymis, prostate, and seminal vesicles from animals infected with either HIV-1_JR-SCF_ or HIV-1_CH040_ (Fig. S6A and B).

**Fig 6:**
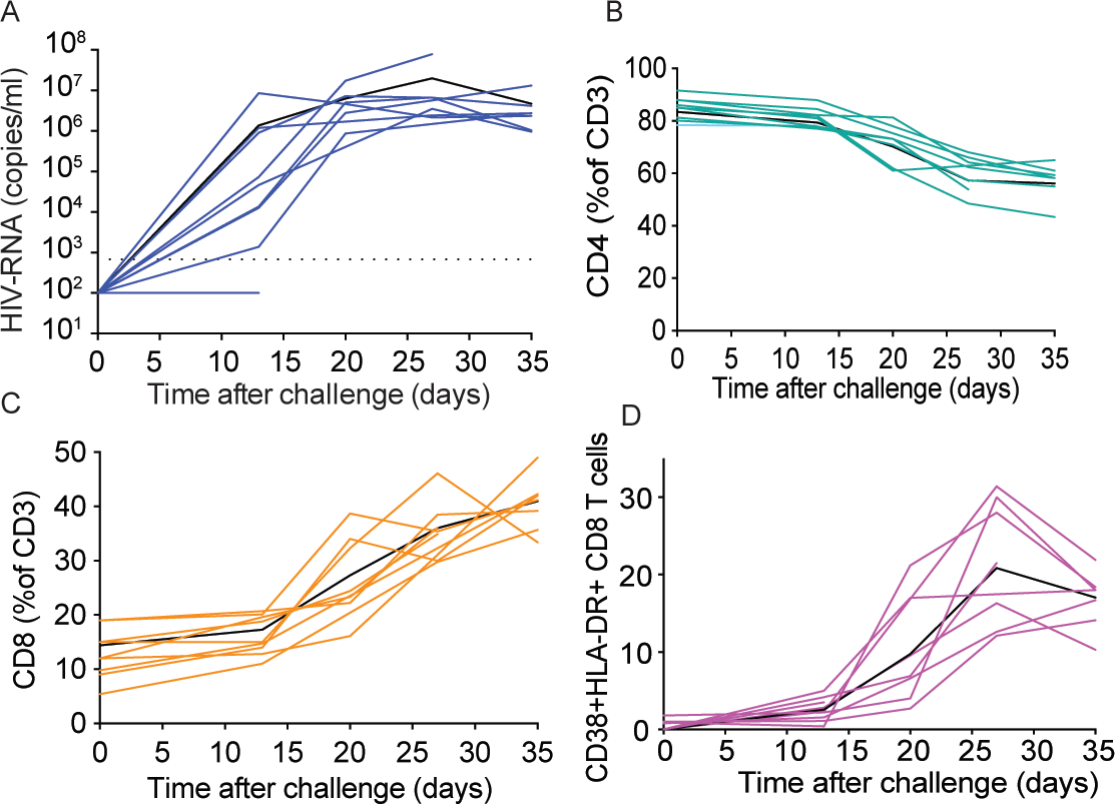
Direct viral exposure to the penis results in systemic HIV infection. Anesthetized male BLT mice were exposed to HIV-1_CH040_ by direct penile exposure onto the meatus urethra. Peripheral blood plasma viral load (**A**), CD4^+^ T cell levels (**B**), CD8^+^ T cell levels (**C**), and CD8^+^ T cell activation levels (**D**) were analyzed longitudinally for 5 weeks. Solid black lines in each panel indicate mean values (*n* = 9).

### HIV infection throughout the MGT is established early after exposure and results in acute CD4^+^ T cell depletion

HIV infection kinetics and its sequelae in the MGT was evaluated in BLT humanized mice (*n* = 13, 8 to 12 weeks posthumanization with 56% human CD45^+^ cells, median 56%, range 26.3–59.2% in peripheral blood). BLT mice were exposed intravenously to HIV-1_JR-CSF_ (3 × 10^4^ TCIU). MGT tissues (testes, epididymis, seminal vesicles, prostate, and penis) and the spleen were collected at 10 (*n* = 5), 20 (*n* = 4), and 60 (*n* = 4) days postexposure for analysis of cell-associated HIV-RNA and CD4^+^ T cell levels (Fig. 5A and B). At 10 day postexposure, 2/8 animals had detectable viremia in peripheral blood plasma. In these animals, cell-associated HIV-RNA was detected in the epididymis, seminal vesicles, prostate, and penis. We did not detect HIV-RNA in any of the tissues analyzed from the six animals without plasma viremia at 10 day postexposure. At 20 and 60 days postexposure, plasma viremia and cell-associated HIV-RNA was detected in all MGT tissues of all BLT mice analyzed (Fig. 5A). At 10 day postexposure, CD4^+^ T cell levels in the MGT from infected animals were similar to those found prior to infection (Fig. 3C and 5B). CD4^+^ T cell levels in the testes, seminal vesicles, prostate, and penis were significantly lower by day 20 postinfection (*P* = 0.0007, 0.0137, 0.0007, 0.001, respectively) (Fig. 5B). In contrast, at day 20 postinfection we did not notice a decrease in the levels of CD4^+^ T cells in the epididymis or the spleen (Fig. 5B. At 60 day postexposure, CD4^+^ T cell levels were significantly lower in the testes (*P* = 0.0001), epididymis (*P* = 0.0002), seminal vesicles (*P* = 0.0001), prostate (*P* = 0.0001), penis (*P* = 0.0001), and spleen (*P* = 0.0001) (Fig. 5B). These results indicate that HIV infection in the MGT is established early after exposure and results in CD4^+^ T cell depletion throughout the entire MGT.

### Direct penile HIV exposure results in systemic viral infection

Worldwide, the vast majority of HIV-positive men acquired HIV infection via heterosexual transmission through the penis (1). Having established that the MGT of BLT mice is reconstituted with human cells that can be productively infected by HIV, we proceeded to determine the susceptibility of BLT mice to HIV infection after atraumatic penile exposure. For this purpose, BLT mice (*n* = 9, human CD45^+^ cells in peripheral blood: median 89.6%, range 62.5–92.7%, CD4 T cells as % of CD3 cells: median 85.1, range 78.4–91.6), were challenged with HIV-1_CH040_ delivered directly to the meatus urethra as indicated in the Materials and methods section. Plasma HIV-RNA, CD4^+^ T cell, CD8^+^ T cell, and T cell activation levels in peripheral blood were monitored longitudinally (Fig. 6). Two weeks post challenge, HIV-RNA was detected in the plasma of 8/9 BLT mice. Peak viremia occurred 4 week post penile exposure (plasma HIV-RNA concentration: median 6.6 × 10^6^, range 2.1 × 10^6^–7.8 × 10^6^ HIV-RNA copies/mL) (Fig. 6A). One animal negative for plasma HIV-RNA was euthanized 14 days postexposure due to health issues; however, cell-associated HIV-DNA was found in the liver, bone marrow, spleen, gut, and MGT from this animal (Table S1) suggestive of the early stages of infection. CD4^+^ T cell levels in the peripheral blood from infected animals started to decline 3 weeks post penile exposure, and this decline became statistically significant at 4 week post challenge (*P* = 0.0025) (Fig. 6B). The levels of activated (CD38^+^HLA-DR^+^) CD8^+^ T cells were highest 4 week postexposure (Fig. 6C and D). In summary, direct penile exposure of BLT mice to a transmitted/founder virus (HIV-1_CH040_) resulted in systemic infection, CD4^+^ T cell depletion, and CD8^+^ T cell activation.

### EFdA effectively reduces HIV levels throughout the entire MGT

**Fig 7:**
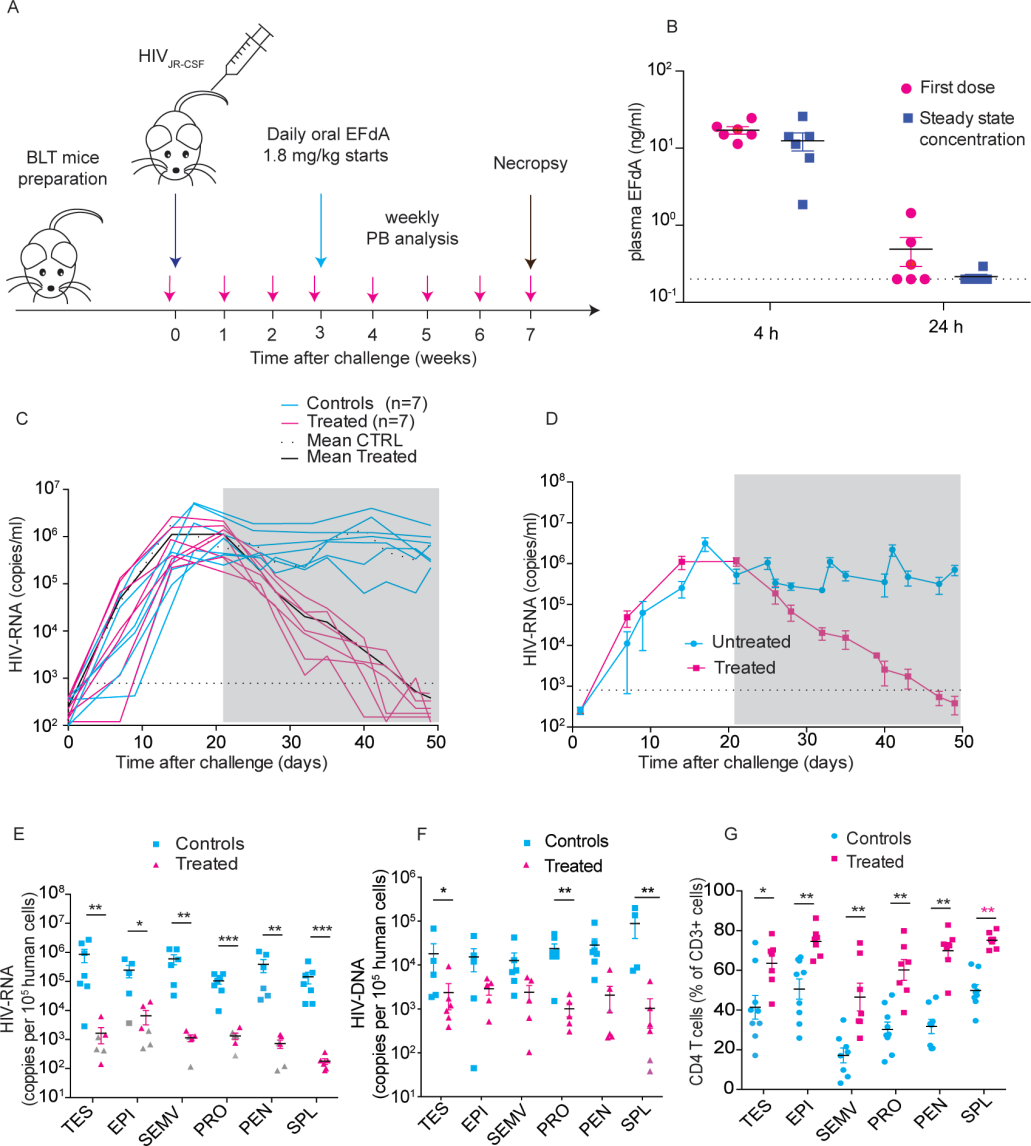
EFdA effectively reduces HIV levels in the peripheral blood and throughout the male genital tract. (**A**) Experimental design. BLT mice (*n* = 14) were exposed intravenously to HIV-1_JR-CSF_. Three weeks post-exposure, infected BLT mice were treated daily with EFdA (1.8 mg/kg) for 4 weeks (*n* = 7) or left untreated (*n* = 7). HIV-RNA in peripheral blood plasma was monitored longitudinally. After 4 weeks of treatment, all mice were euthanized and cell-associated HIV-RNA, cell-associated HIV-DNA and CD4^+^ T cell levels in the tissues of the MGT were analyzed. (**B**) Analysis of EFdA levels in plasma 4 hour and 24 hour after the first oral dose (left) and at steady state after 1 week of daily oral dosing (mean ± s.e.m. are shown). (**C**) HIV-RNA levels in the plasma of individual mice. (**D**) Mean plasma viremia (± s.e.m.) of the EFdA-treated and control animals (**C**). The grey shaded area indicates the time of EFdA treatment. The dotted line indicates the HIV-RNA limit of detection. Analysis of cell-associated HIV-RNA (**E**), cell-associated HIV-DNA (**F**), and CD4^+^ T cell levels (**G**) in MGT tissues and the spleen 4 weeks after initiation of treatment with EFdA. The HIV-RNA limit of detection (LOD) was calculated for each sample based on the number of human cells available for analysis for each tissue. Grey symbols in (**E**), (**F**) and (**G**) indicate samples below the LOD and black lines indicate mean values for each data set. A Mann–Whitney U test was used to compare cell-associated HIV-RNA (**E**), cell-associated HIV-DNA (**F**) and CD4 T cell levels. (*P < 0.05, **P < 0.01, ***P < 0.001, *n* = 9).

Decreased viral load in blood and semen results in a dramatic reduction of HIV transmission (33). To assess the efficacy of antiretroviral drugs to suppress HIV infection in the MGT, male BLT mice were exposed to HIV-1_JR-CSF_ intravenously (*n* = 14, peripheral blood human CD45^+^ cells: median 62.3%, range 44.9–74.4%; CD4^+^ T cells as % of CD3^+^ cells: median 82%, range 96.2–87.9). Three weeks later, infected BLT mice were divided into two groups with similar levels of human cells and plasma viral loads (*n* = 7 each, *P* = 0.7154, and *P* = 0.9656, respectively). To obtain proof of principle, one group was treated with EFdA (4′-ethynyl-2-fluoro-2′-deoxy-adenosine, Islatravir), a nucleoside reverse transcriptase translocation inhibitor (NRTTI), (*n* = 7, 1.8 mg /kg/day, equivalent of single human 10 mg dose (66)) administered orally for 4 weeks. The second group of mice (*n* = 7) did not receive treatment and served as an untreated control (Fig. 7A). The presence of EFdA in plasma was confirmed 4 and 24 hours after the first oral dose and 4 and 24 hours after 1 week of daily oral dosing (Fig. 7B). The median plasma concentration of EFdA at 4 hour postadministration of the first dose was 16.3 ng/mL, and this concentration was not significantly different from the steady state concentration 4 hours postadministration after 1 week of daily treatment (12.7 ng/mL, *P* = 0.1190). Similarly, the EFdA concentration at 24 hour postadministration of the first dose was not significantly different from the steady state concentration at 24 hour postadministration after 1 week of daily treatment (0.255 ng/mL vs. 0.2 ng/mL, *P* = 0.1818) (Fig. 7B). EFdA treatment resulted in a rapid and progressive decrease in the plasma viral load (Fig. 7C and D). After 4 week of drug treatment, the levels of plasma viremia in all BLT mice were below the limit of detection (687.5 copies/mL plasma). At the same time, control untreated mice had mean HIV-RNA levels of 7 × 10^5^ copies/mL. Tissues from these mice were harvested and processed for the analysis of cell-associated HIV-RNA and HIV-DNA. EFdA treatment resulted in a 3-log reduction in cell-associated HIV-RNA levels in the testes, seminal vesicles, and penis and in a 2-log reduction in the HIV-RNA levels present in the epididymis and the prostate compared to the levels of HIV-RNA in the tissues from untreated control animals (Fig. 7E). Notably, cell-associated HIV-RNA could not be detected in multiple MGT tissue samples obtained from EFdA-treated mice (Fig. 7E). Cell-associated HIV-DNA levels in EFdA-treated mice significantly decreased in the testes, prostate, and spleen (Fig. 7F). Treatment with EFdA also resulted in significant increases in CD4^+^ T cell levels in all MGT tissues compared to untreated control animals (Fig. 7G). Therefore, EFdA treatment not only dramatically reduced the levels of viral replication in the MGT, but it also resulted in a strong recovery of CD4^+^ T cells levels throughout the entire MGT (Fig. 7G).

### EFdA treatment offers significant protection from penile HIV infection

**Fig 8:**
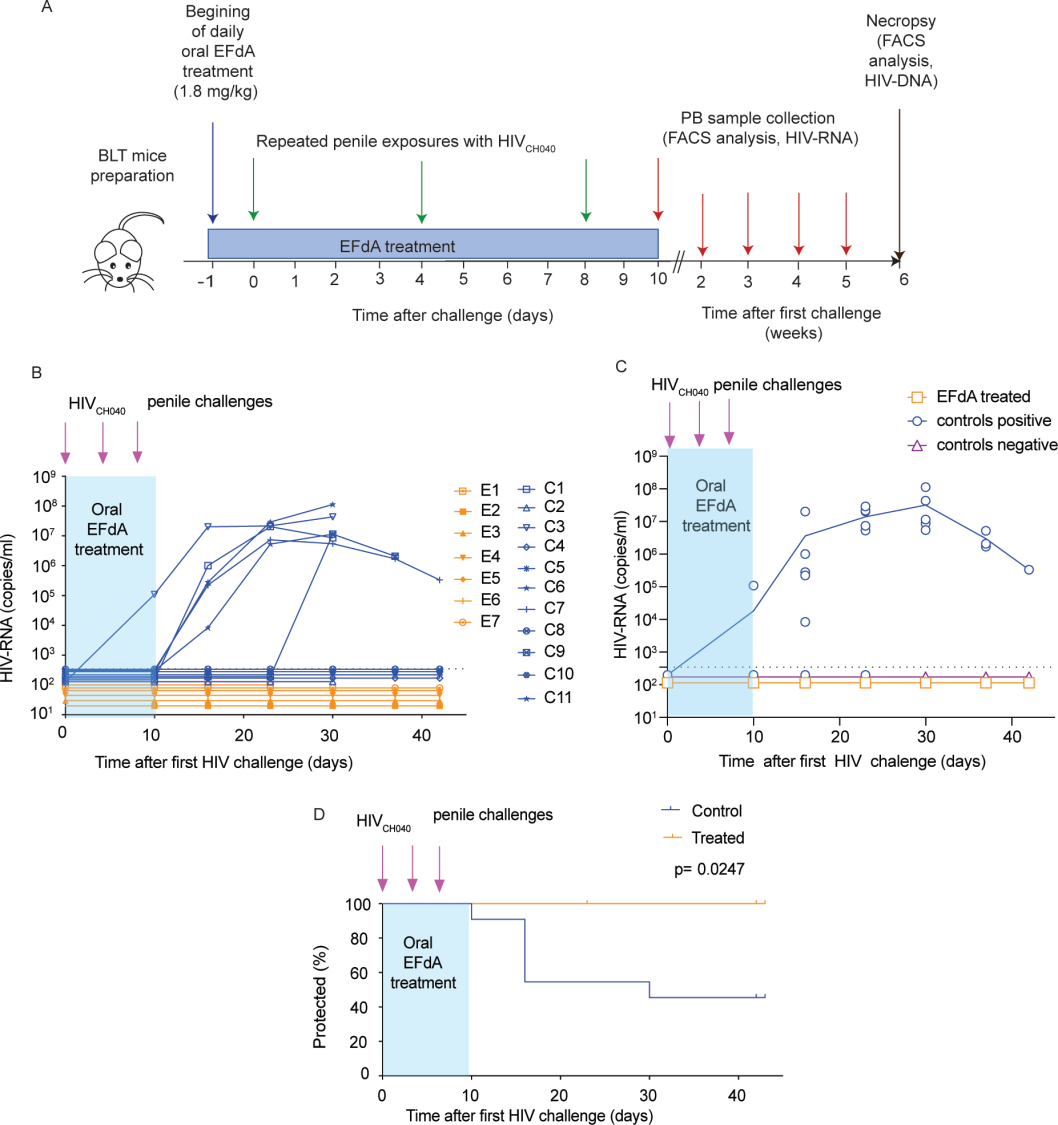
EFdA protects against penile HIV transmission. (**A**) Experimental design. Untreated BLT mice (*n* = 11) and BLT mice treated daily with oral EFdA (1.8 mg/kg) (*n* = 7) were challenged with HIV-1_CH040_ via their penis at days 1, 4 and 8 of treatment. Plasma HIV-RNA levels in peripheral blood were monitored longitudinally. (**B**) Plasma level of HIV-RNA in individual mice, (**C**) the mean HIV-RNA levels in the plasma of control untreated mice (blue line) or EFdA treated mice (orange line) with circles indicating the HIV-RNA values for each individual mouse. For clarity of representation in the figure, the HIV-RNA values for control uninfected mice and EFdA-treated mice with undetectable HIV-RNA in plasma were inputted as 1/2 and 1/3 of the limit of detection (346.3 copies/mL), respectively. (**D**) Kaplan–Meier plot showing significant protection from penile HIV transmission in EFdA-treated BLT mice compared to untreated controls. The blue shaded area indicates the time of daily oral treatment with EFdA. The dotted line in B, C indicates the HIV-RNA limit of detection.

The potential of EFdA to prevent penile HIV acquisition was evaluated in BLT humanized mice. BLT mice treated daily with EFdA (1.8 mg/kg/day, *n* = 9) and untreated controls (*n* = 11) were exposed to HIV-1_CH040_ via the penis 1, 4, and 8 days after treatment initiation. Two days after the final challenge (day 10 of treatment), EFdA treatment was discontinued, and the levels of HIV-RNA in plasma analyzed at that time and then weekly thereafter (Fig. 8A).

Whereas none of the EFdA treated animals became positive for HIV-RNA in plasma, of the control untreated mice, one mouse became HIV positive 10 days after the first exposure, four mice became positive 2 week after the first exposure, and one mouse at 4 week after the first exposure (Fig. 8B and C). Six weeks after the first HIV challenge, mice were necropsied and multiple tissues were analyzed for the presence of cell-associated HIV-DNA. Cell-associated DNA was identified in tissues of all mice with detectable HIV-RNA in plasma. No evidence of cell-associated HIV-DNA was noted in any of the tissues analyzed from mice with undetectable HIV-RNA in plasma. In total, 6 out of 11 control untreated mice had detectable HIV-RNA in plasma and cell-associated HIV-DNA in tissues. In contrast, none of the EFdA-treated mice were positive for HIV-RNA in plasma or HIV-DNA in tissues, indicating full systemic protection from HIV infection (Fig. 8D, Table S2, *P* = 0.0247, Mantel–Cox log rank test).

## DISCUSSION

In this manuscript, we used an *in vivo* model that faithfully recapitulates key features of HIV infection in the MGT and that permits the detailed analysis of all the individual organs of the MGT to show that (i) all MGT tissues are reconstituted with human hematopoietic cells, (ii) the human cells present in the MGT are predominantly T cells expressing CCR5, (iii) cells present throughout the entire MGT are productively infected with HIV regardless of the route of inoculation or viral isolate, and (iv) HIV infection results in CD4^+^ T cell depletion of MGT tissues. Furthermore, we used this model to show that HIV infection in MGT tissues can be effectively treated with antiretroviral therapy resulting in a dramatic reduction in viral load and restoration of CD4^+^ T cell levels throughout the entire MGT (Fig. 7). We also show that oral preexposure prophylaxis efficiently protects from HIV acquisition after penile HIV exposure (Fig. 8).

Recently, HIV/SIV chimeric viruses are becoming more common and provide opportunities to explore how transmitter/founder virus envelopes contribute to acquisition in non-human primates. Due to the extremely limited tropism of HIV, humanized mice containing an *in situ* generated human immune system are an ideal model for the evaluation of ARV efficacy that permits the utilization of clinically relevant HIV strains. However, the appropriate distribution and tissue localization of the human cells present throughout different MGT tissues is important for modelling HIV infection. The MGT tissues of BLT mice with the highest levels of human cells were the prostate, seminal vesicles, and penis (glans). Importantly, similarly to what is observed in the MGT of humans and macaques (36, 67 - 69), human CD4^+^ T cells were found located in close proximity to the epithelium and stroma of the different MGT tissues (Fig. 1 and 2). It should be noted that we did not quantitatively analyze interspecies differences in hematopoietic cells in tissues of MGT. Human myeloid cells (hCD68+) were also observed in all MGT compartments; however, they were significantly less abundant than T cells (36, 67 - 69). Consistent with these low numbers of myeloid cells, we were not able to identify infected macrophages in the MGT of BLT humanized mice at 2 or 6 weeks of systemic HIV infection. Our analysis indicated a low number of myeloid cells in the different tissues of MGT in this model. Although tissue macrophages play an important role in HIV persistence as they can harbor HIV reservoirs, T cells are important drivers of HIV transmission. Thus, we do not expect that the lower number of myeloid cells in MGT tissue would underestimate the drug dose needed to prevent HIV transmission.

Despite the fact that the MGT of humans and mice have similar anatomical and functional roles some differences exist (70). The mouse prostate consists of four paired lobes while the human prostate is categorized by zones. The mouse penis has a male urogenital mating protuberance (MUMP), and a penile bone (os penis) that are absent in humans. The foreskin (prepuce) plays an important role in penile transmission in humans as evidenced by a 40–60% decrease in penile transmission in circumcised men (33). In contrast to humans, the mouse penis has an internal and an external foreskin. The external foreskin tissue is composed of normal hair-bearing skin epithelial tissue. The internal prepuce is a typical mucosal epithelium and tightly covers the glans (71). Notwithstanding those differences, penile exposure to a T/F HIV in BLT mice resulted in successful systemic HIV infection (Fig. 6). Due to the small size of the mouse penis, the HIV inoculum was administered to the tip of the penis of anesthetized BLT mice providing exposure of the glans, urethral epithelium, and mucosal internal foreskin to virus. Slight pressure on the mouse lower abdomen resulted in erection of the penis and allowed application of the virus inoculum to the tip of the penis; therefore, the procedure was not invasive. Even though we took all reasonable precautions to perform an atraumatic exposure, we cannot rule out the possibility (albeit small) that infection could have taken place through a microscopic rupture of blood capillaries. The similarities in the location of hematopoietic cells and the phenotype of the CD4^+^ T cells present in the MGT of BLT mice with those in humans, and the susceptibility of BLT mice to HIV infection following penile exposure highlights the utility of BLT mice as an *in vivo* model for this route of HIV infection. The newly established model was therefore used to obtain proof of principle that EFdA, a drug currently in clinical development, can suppress viremia throughout the entire MGT and prevent HIV acquisition via the penis.

EFdA is a highly effective NRTTI under evaluation in clinical trials for the treatment of HIV infection (56, 72 - 75). It is a deoxyadenosine analog with a chemical structure that leads to (i) very high affinity to the reverse transcriptase, (ii) blocking of primer translocation, and (iii) immediate chain termination of transcription, and an extended half-life (72 - 74). These multiple mechanisms of action, coupled with its high binding affinity to reverse transcriptase, translate into robust antiviral efficacy, making it the most potent NRTTI described to date (72 - 74). Previously we showed that daily treatment of BLT mice with EFdA efficiently prevented vaginal or oral HIV transmission (56). We also demonstrated that EFdA treatment of infected BLT mice resulted in a significant reduction of HIV-RNA levels in plasma and strong systemic suppression of HIV replication (75).

We evaluated the ability of EFdA to effectively suppress chronic HIV infection in each individual MGT tissue. HIV in semen, which is the primary vector for sexual HIV transmission, is derived from both blood plasma and various tissues within the MGT (3, 32, 76, 77). Although treatment of HIV infection requires long-term multi-drug regimens to prevent the development of HIV resistance and treatment failure, the ability of a single drug to efficiently suppress HIV infection in all analyzed tissues of the MGT within a short period of time indicates sufficient penetration of the drug to the MGT and suggests a high potential for the drug in HIV treatment. Importantly, HIV suppression in MGT tissues was accompanied with strong CD4^+^ T cell recovery and repopulation in all MGT tissues analyzed (Fig. 7G). This is a critical observation as clinical studies evaluating EFdA for the treatment and prevention of HIV infection were recently placed on hold by the FDA based on a decrease in total lymphocyte and CD4^+^ T cell counts in some participants (78). The fact that HIV infection can be efficiently suppressed by EFdA and that this suppression is accompanied by human CD4^+^ T cell recovery have broad implications for the adjustment of human dosing regimens to improve EFdA safety.

Sexually transmitted HIV infections in exclusively heterosexual men are acquired through the penis. Low adherence to condom usage and the limited protection provided by circumcision requires additional methods for protection from penile HIV transmission. Preexposure prophylaxis is an important mode of HIV prevention that can be achieved with a single drug regimen (79). We were able to demonstrate the efficacy of EFdA to prevent HIV acquisition after penile exposure. It should be noted that a high HIV dose was used in the prevention efficacy experiment to achieve HIV transmission in most experimental animals. This also increased the stringency of the model. In further refinements of the model, it might be possible to perform low dose repeated exposure experiments. However, this will have to be determined empirically in future experiments. Together, our preclinical data suggests that EFdA is an effective ARV capable of suppressing HIV replication through the entire MGT and preventing penile HIV acquisition.

## SUPPLEMENTAL MATERIAL

FIG S1 mbio.02224-22-s0001.jpg Immunohistochemical control staining of penile tissues for images in Fig. 1.

FIG S2 mbio.02224-22-s0002.jpg Immunohistochemical control staining of MGT tissues for images in Fig. 2.

Fig S3 mbio.02224-22-s0003.jpg Flow cytometry analysis of macrophages in the MGT.

Fig S4 mbio.02224-22-s0004.jpg FACS analysis of CCR5 expression in human CD4 T cells.

Fig S5 mbio.02224-22-s0005.jpg Immunofluorescence analysis for HIV infection in human CD68 cells.

Fig S6 mbio.02224-22-s0006.jpg Cell-associated and cell-free HIV-RNA levels in indicated tissues of the MGT.

Supplementary Material 1 mbio.02224-22-s0007.docx Supplemental tables and figure legends.

ASM does not own the copyrights to Supplemental Material that may be linked to, or accessed through, an article. The authors have granted ASM a non-exclusive, world-wide license to publish the Supplemental Material files. Please contact the corresponding author directly for reuse.
