# Supplementary figures and images for: EFdA efficiently suppresses HIV replication in the male genital tract and prevents penile HIV acquisition
Source: mBio. 2023 Jun 12;14(4):e02224-22. doi: 10.1128/mbio.02224-22 (PMC10470584; doi:10.1128/mbio.02224-22)

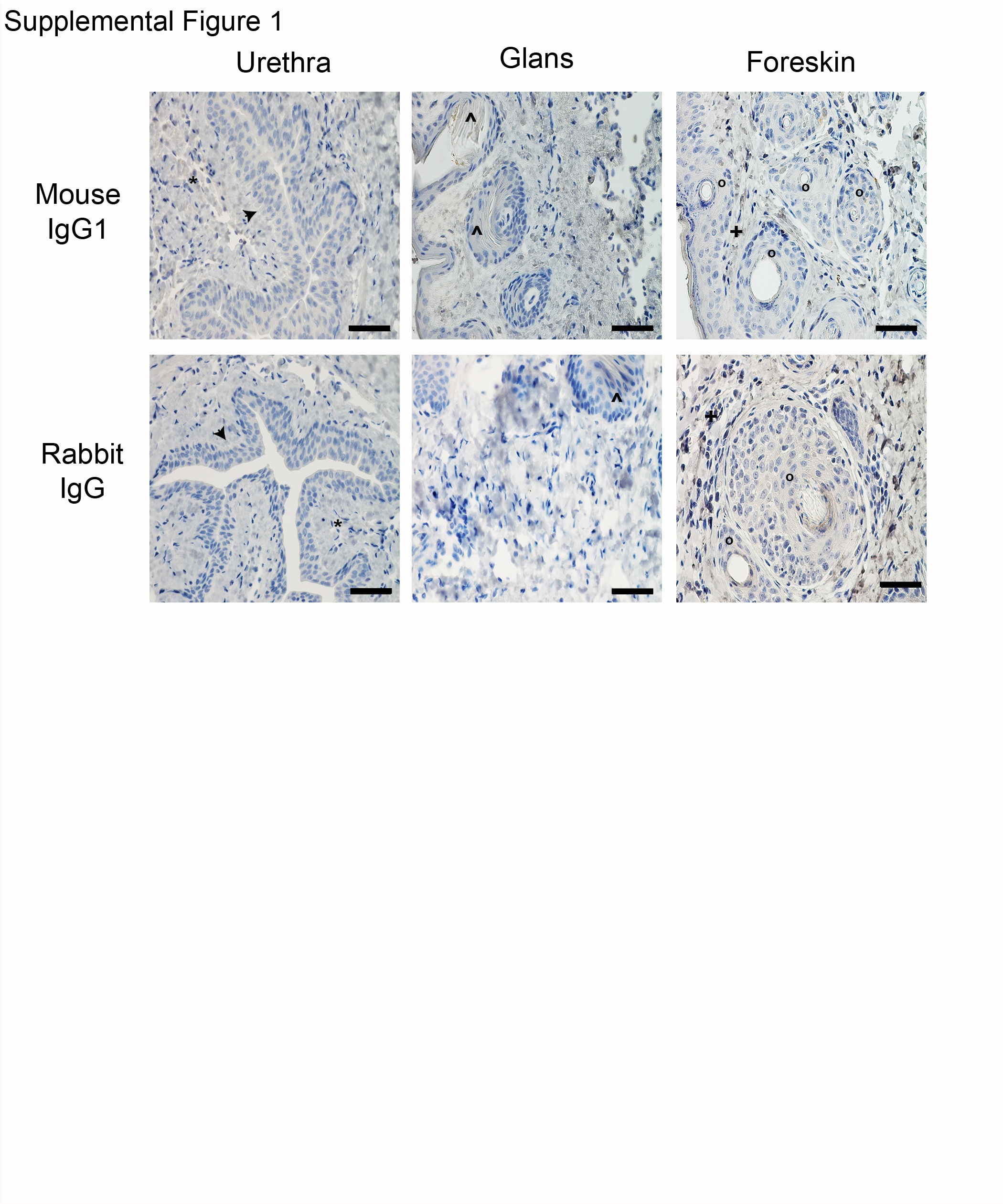

Supplement: FIG S1 — Immunohistochemical control staining of penile tissues for images in Fig. 1. [file mbio.02224-22-s0001.jpg]

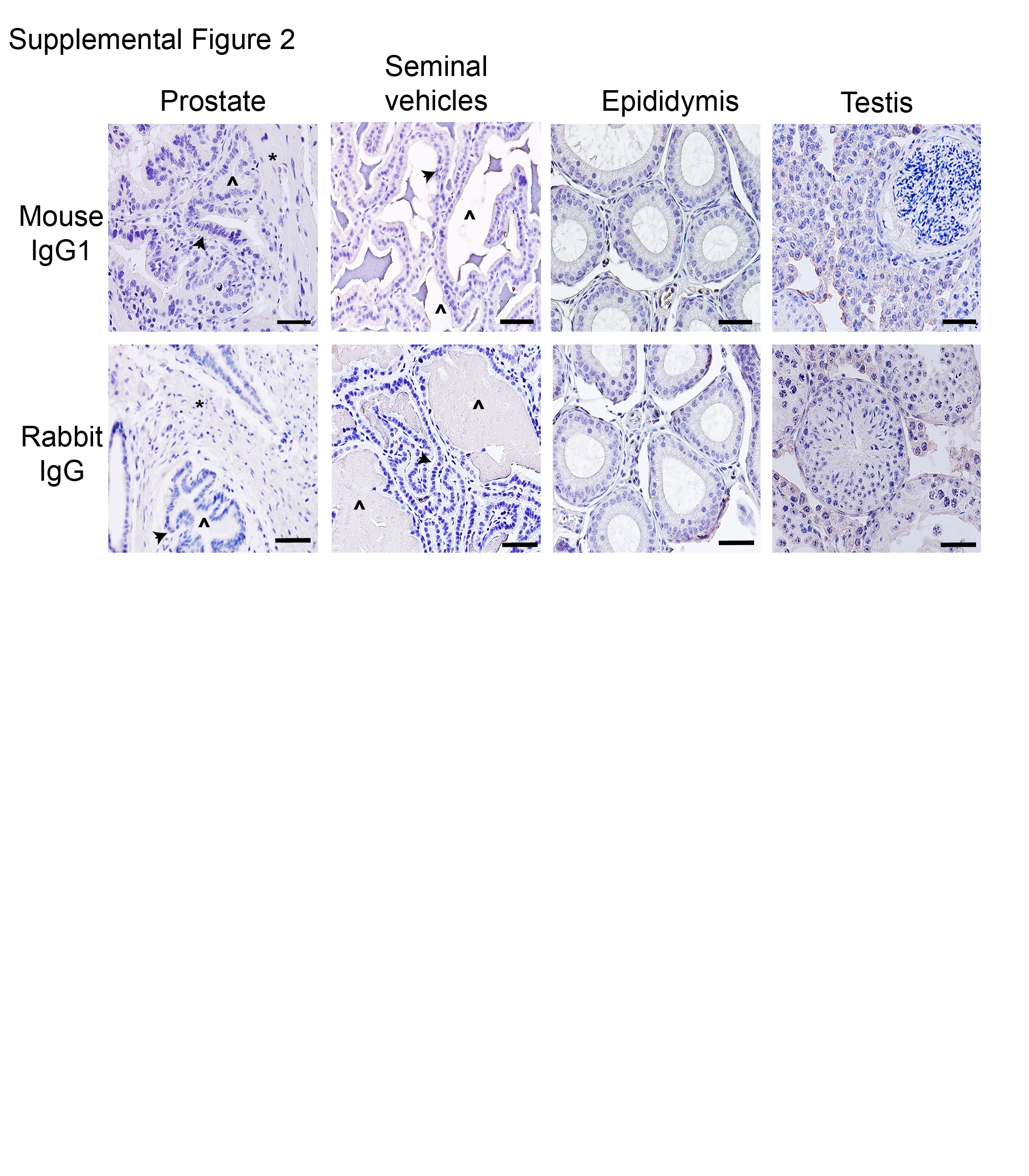

Supplement: FIG S2 — Immunohistochemical control staining of MGT tissues for images in Fig. 2. [file mbio.02224-22-s0002.jpg]

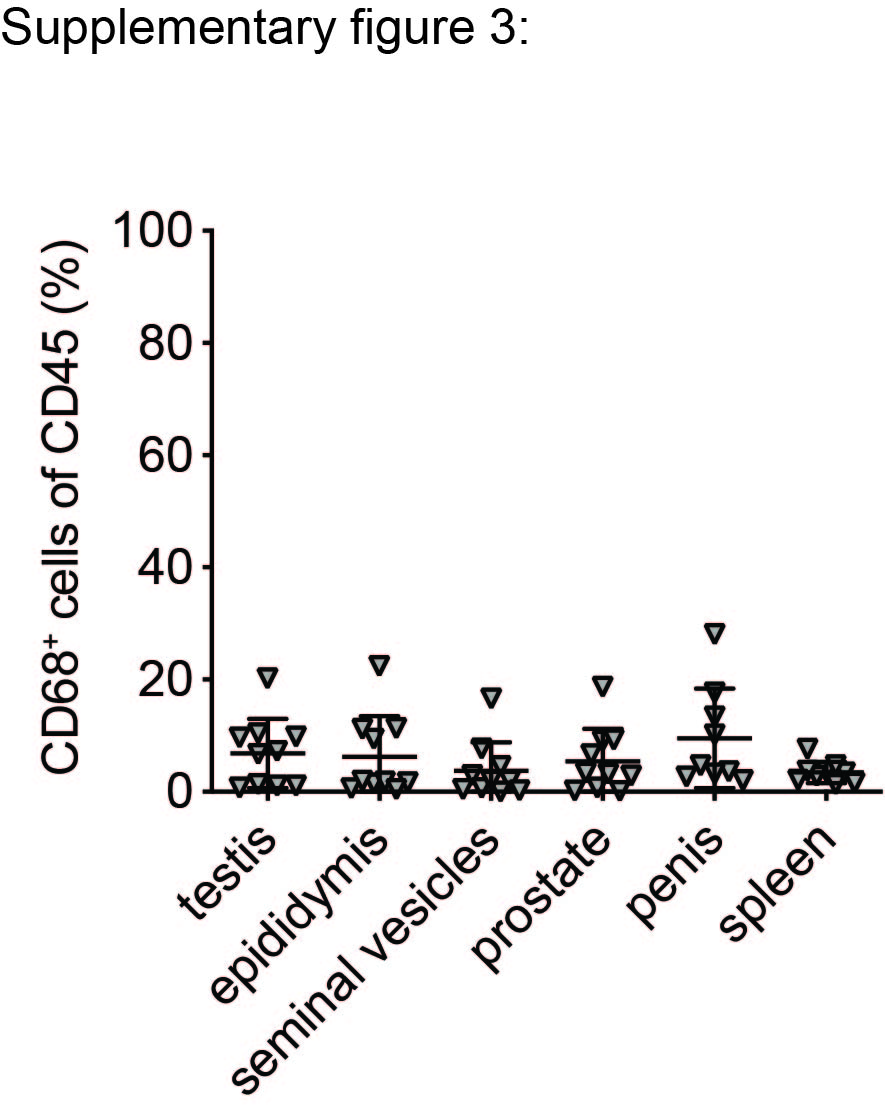

Supplement: Fig S3 — Flow cytometry analysis of macrophages in the MGT. [file mbio.02224-22-s0003.jpg]

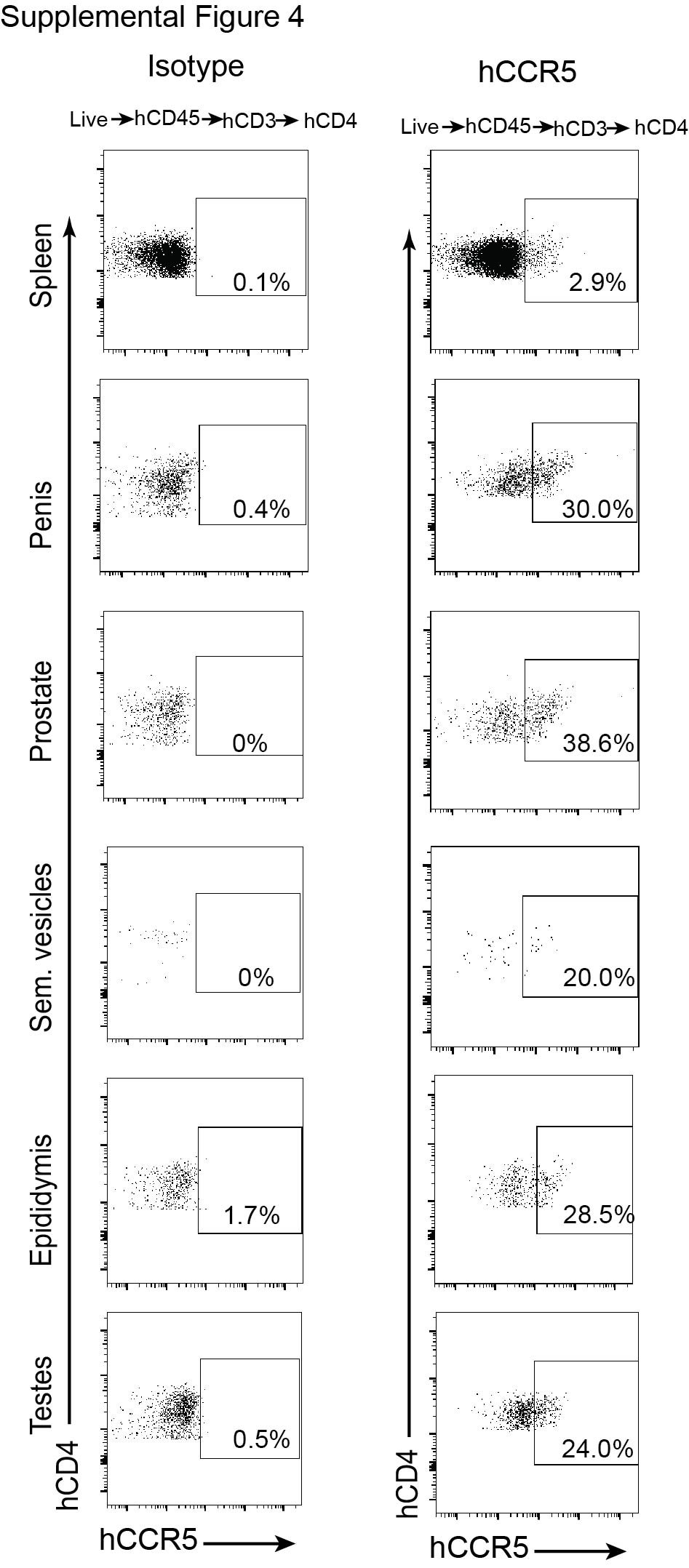

Supplement: Fig S4 — FACS analysis of CCR5 expression in human CD4 T cells. [file mbio.02224-22-s0004.jpg]

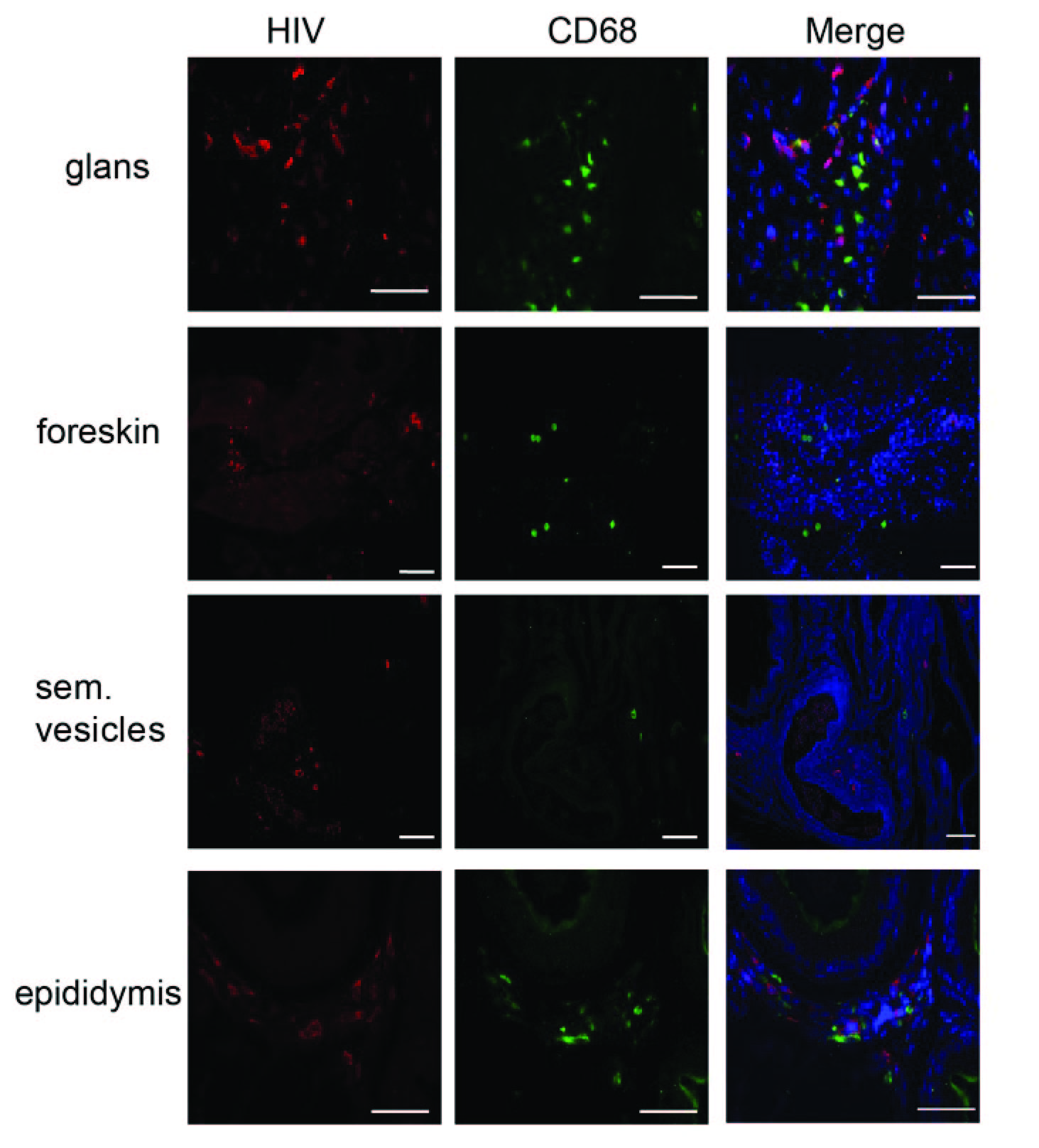

Supplement: Fig S5 — Immunofluorescence analysis for HIV infection in human CD68 cells. [file mbio.02224-22-s0005.jpg]

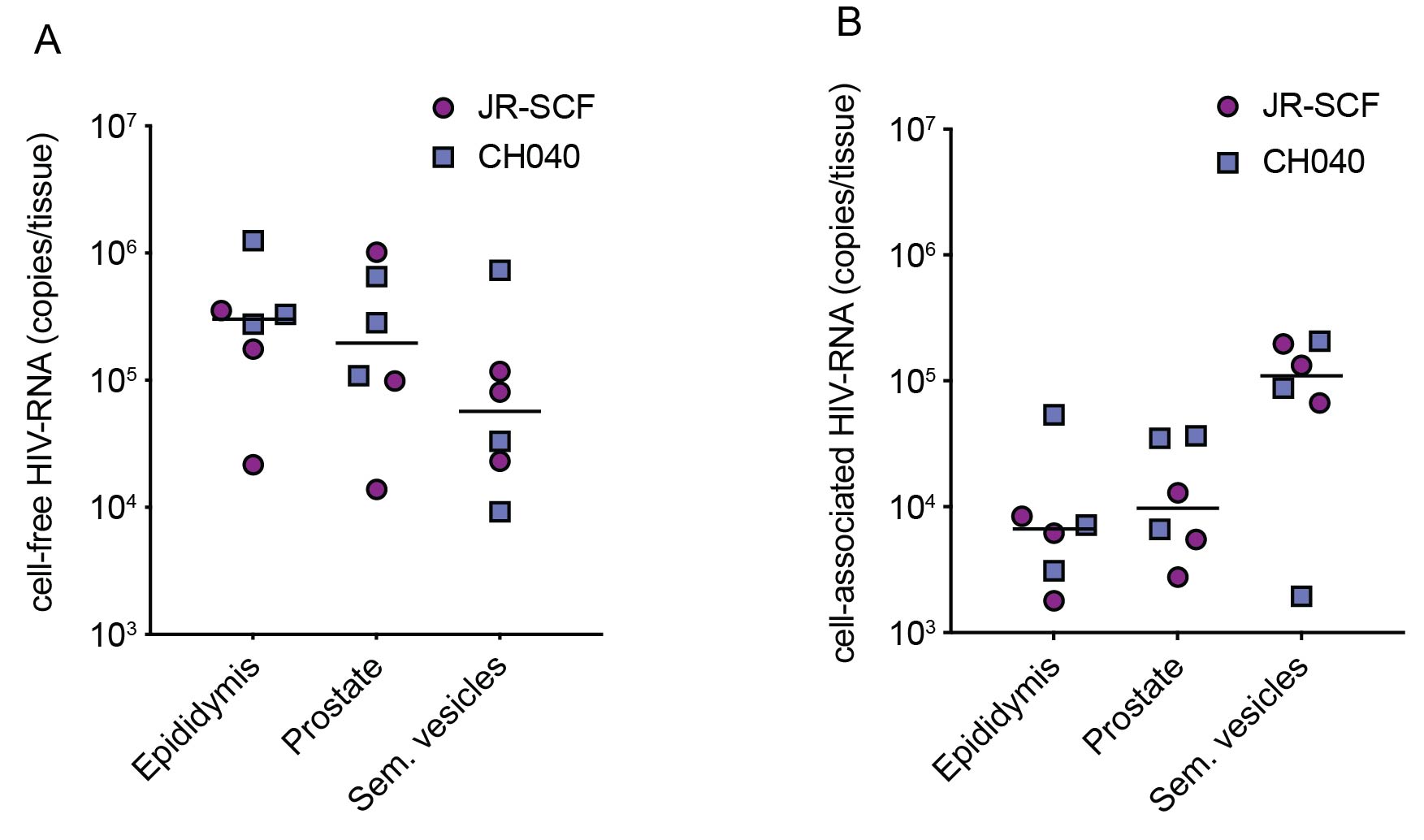

Supplement: Fig S6 — Cell-associated and cell-free HIV-RNA levels in indicated tissues of the MGT. [file mbio.02224-22-s0006.jpg]
