# Supplementary material for: EFdA efficiently suppresses HIV replication in the male genital tract and prevents penile HIV acquisition
Source: mBio. 2023 Jun 12;14(4):e02224-22. doi: 10.1128/mbio.02224-22 (PMC10470584; doi:10.1128/mbio.02224-22)
Supplement: Supplementary Material 1 — Supplemental tables and figure legends. [file mbio.02224-22-s0007.docx]

**Supplemental material:**

**Table S1:** **Cell-associated HIV-DNA in BLT mice exposed to HIV via penis (Figure 6).**

BM: bone marrow, Org: Thymic organoid, LN: lymph nodes, IEL: Intraepithelial lymphocytes, LPL: Lamina propria lymphocytes, + presence of cell-associated HIV-DNA, - cell-associated HIV-DNA bellow limit of detection, n.a. cell-associated HIV-DNA not analyzed.

**Table S2: Cell-associated HIV-DNA in BLT mice treated with EFdA as pre-exposure prophylaxis to prevent penile HIV transmission. (Figure 8).**

Mice E1-E7 were treated with EFdA (1.8 mg/kg), mice C1-C11 are untreated controls. BM: bone marrow, Org: Thymic organoid, LN: lymph nodes, + presence of cell-associated HIV-DNA, - cell-associated HIV-DNA bellow limit of detection, n.a. cell-associated HIV-DNA not analyzed.

**Figure S1: Immunohistochemical control staining of penile tissues for images in the Figure 1.**

Sections of urethra, glans, and foreskin from a representative male BLT mouse were stained with an isotype control for anti-human CD45 and anti-human CD68 antibodies (mouse IgG1) or rabbit IgG, an isotype for the anti-human CD3 and anti-human CD4 antibodies. Urethral epithelium (arrowheads), lamina propria (*), keratinized penile spines (^), foreskin epithelium (+), hair follicles in foreskin (°). Note the absence of brown staining. Scale bars represent 50 μm. Tissue sections shown are representative of 12 sections taken from 6 uninfected BLT mice.

**Figure S2: Immunohistochemical control staining of male genital track tissues for images in the Figure 2.**

Sections of prostate, seminal vesicles, epididymis, and testis from a male BLT mouse were stained with an isotype control for anti-human CD45 and anti-human CD68 antibodies (mouse IgG1) or rabbit IgG, isotype for anti-human CD3 and anti-human CD4 antibodies. Note the absence of brown staining. Epithelium (arrowheads), smooth muscle (*), gland lumen (^). Scale bars represent 50 μm. Tissue sections shown are representative of 12 sections taken from 6 uninfected BLT mice.

**Figure S3: Flow cytometry analysis of macrophages in the MGT.** Cells isolated from spleen, penis, prostate, seminal vesicles, epididymis, and testes of uninfected male BLT mice (n=10) were analyzed by flow cytometry to evaluate the levels of human macrophages in each compartment. Levels of macrophages are shown as a percentage of the total CD45 cells present.

**Figure S4: FACS analysis of CCR5 expression in human CD4 T cells.**

Mononuclear cells isolated from indicated tissues of MGT of BLT mice were stained with hCD45, hCD3, hCD4, and hCCR5 or its isotype control and analyzed by FACS. Representative dot plots of CD4 T cells from spleen, penis, prostate, seminal vesicles, epididymis, and testes stained with hCCR5 or its isotype are shown.

**Figure S5: Immunofluorescence analysis for HIV infection in human CD68^+^ cells.**

Double immunofluorescence staining analysis for HIVp24 (red) and human CD68 (green) expression. Representative images of epididymis, seminal vesicles, glans and foreskin from a male BLT mouse 14 days post intravenous infection with HIV_CH040_. DAPI (shown in blue) was used as a counterstain for nucleated cells. The scale bars represent 50 μm. Sections shown are representative of 6 sections taken from 3 infected BLT mice.

**Figure S6:**  **Cell-associated and cell-free HIV-RNA levels in indicated tissues of the MGT.** Male BLT mice were infected intravenously with either HIV_JR-CSF_ (n=3) or HIV_CH040_ (n=3). Two weeks post infection mice were euthanized, and epididymis, prostate, and seminal vesicles were analyzed for cell-associated (A) and cell-free (B) HIV-RNA. The quantity of HIV-RNA was calculated per whole organ/tissue to enable comparison between cell-associated and cell free virus. Lines indicate median value for each group.
